# Opioid and neuroHIV Comorbidity – Current and Future Perspectives

**DOI:** 10.1007/s11481-020-09941-8

**Published:** 2020-09-02

**Authors:** Sylvia Fitting, MaryPeace McRae, Kurt F. Hauser

**Affiliations:** 1 Department of Psychology and Neuroscience, University of North Carolina at Chapel Hill, Chapel Hill, NC 27599-3270 USA; 2 Department of Pharmacotherapy and Outcomes Science, School of Pharmacy, Virginia Commonwealth University, Richmond, VA 23298 USA; 3 Department of Pharmacology and Toxicology, School of Medicine, Virginia Commonwealth University, 1217 East Marshall Street, Richmond, VA 23298-0613 USA; 4 Department of Anatomy and Neurobiology, School of Medicine, Virginia Commonwealth University, Richmond, VA 23298-0709 USA; 5 Institute for Drug and Alcohol Studies, Virginia Commonwealth University, 203 East Cary Street, Richmond, VA 23298-0059 USA

**Keywords:** Antiretroviral therapy, Astrocyte, Blood-brain barrier, Buprenorphine, C-C motif chemokine receptor 5 (CCR5), COVID-19, Cytochrome P450 3A4 (CYP 3A4), Endogenous opioid system of peptides and receptors, Functional selectivity/biased agonism, HIV-associated neurocognitive disorders, Maladaptive neuroplasticity, Methadone, Microglia, μ-Opioid receptor (*OPRM1*), neuroHIV, Oligodendroglia, P-glycoprotein, Pro-brain-derived neurotrophic factor (pro-BDNF), Synaptodendritic degeneration

## Abstract

With the current national opioid crisis, it is critical to examine the mechanisms underlying pathophysiologic interactions between human immunodeficiency virus (HIV) and opioids in the central nervous system (CNS). Recent advances in experimental models, methodology, and our understanding of disease processes at the molecular and cellular levels reveal opioid-HIV interactions with increasing clarity. However, despite the substantial new insight, the unique impact of opioids on the severity, progression, and prognosis of neuroHIV and HIV-associated neurocognitive disorders (HAND) are not fully understood. In this review, we explore, in detail, what is currently known about mechanisms underlying opioid interactions with HIV, with emphasis on individual HIV-1-expressed gene products at the molecular, cellular and systems levels. Furthermore, we review preclinical and clinical studies with a focus on key considerations when addressing questions of whether opioid-HIV interactive pathogenesis results in unique structural or functional deficits not seen with either disease alone. These considerations include, understanding the combined consequences of HIV-1 genetic variants, host variants, and μ-opioid receptor (MOR) and HIV chemokine co-receptor interactions on the comorbidity. Lastly, we present topics that need to be considered in the future to better understand the unique contributions of opioids to the pathophysiology of neuroHIV.

**Graphical Abstract:**
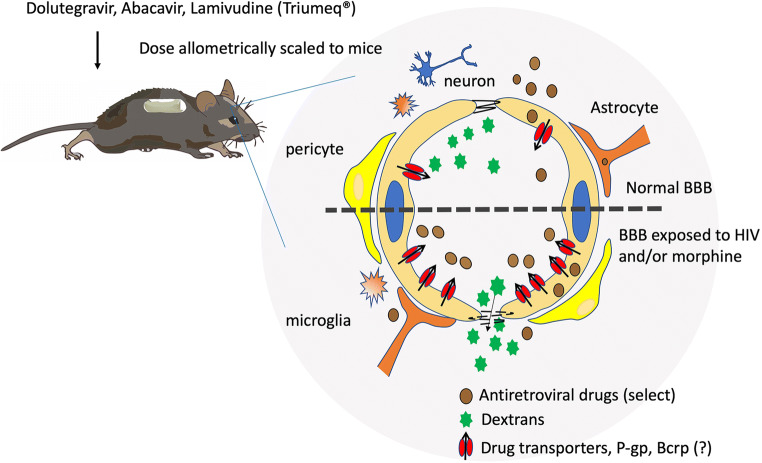
Blood-brain barrier and the neurovascular unit. With HIV and opiate co-exposure (represented below the dotted line), there is breakdown of tight junction proteins and increased leakage of paracellular compounds into the brain. Despite this, opiate exposure selectively increases the expression of some efflux transporters, thereby restricting brain penetration of specific drugs.

Blood-brain barrier and the neurovascular unit. With HIV and opiate co-exposure (represented below the dotted line), there is breakdown of tight junction proteins and increased leakage of paracellular compounds into the brain. Despite this, opiate exposure selectively increases the expression of some efflux transporters, thereby restricting brain penetration of specific drugs.

## Overview

### The Opioid Crisis

Opioid abuse in the United States (U.S.) has reached catastrophic levels. According to the latest World Drug Report, 53.4 million people worldwide used opioids in 2017, which is 56% higher than in the previous year (UNODC 2019a). North America remains the region with the highest non-medical use of opioids with a staggering 4% of the population aged 15–64 using opioids (UNODC 2019a, c). In 2017, the burden of opioid use in the U.S. had accounted for 42 million healthy years of life lost due to premature death and disability (Institute for Health Metrics and Evaluation 2017; UNODC 2019a). During 2017, there were 70,237 overdose related deaths, out of which 47,600 (67.8%) were caused by opioids, which was a 12% increase from 2016 (Scholl et al. 2018; UNODC 2019c). The Centers of Disease Control and Prevention (CDC) reports that on average 130 Americans die from an opioid overdose each day (CDC 2017). Due to the constant rise in deaths involving opioids, the U.S. Government declared the opioid crisis/epidemic a public health emergency in 2017 (U.S. Department of Health and Human Services 2017). Injection drug use increases the likelihood of contracting human immunodeficiency virus (HIV) and drug abuse and HIV have long been described as interrelated epidemics (Swan 1997; Leshner 1998; Nath et al. 1999, 2002). Despite this understanding, opioid use disorder (OUD) and HIV remain a huge public health concern (Strathdee and Beyrer 2015; Peters et al. 2016). In fact, the opioid crisis is seen as a major roadblock in several aspects of public health, including thwarting the goal of eliminating HIV within the next decade (Fauci et al. 2019; Lerner and Fauci 2019).

OUD is also likely to exacerbate many negative aspects of the COVID-19 pandemic (Alexander et al. 2020; Becker and Fiellin 2020; NIDA 2020; Wakeman et al. 2020). Not only are individuals with OUD more vulnerable to SARS-CoV-2 and liable to spread the infection, social-distancing practices create isolation, despair, and economic hardships, heightening opioid abuse (with inherent respiratory depression depending on the amount of tolerance developed) and the probability of overdose (Becker and Fiellin 2020; Wakeman et al. 2020). By virtue of its greater safety profile and decreased likelihood for abuse (Bell and Strang 2020), the use of buprenorphine via telemedicine has become advantageous for managing OUD during the COVID-19 pandemic (Leppla and Gross 2020; Samuels et al. 2020) but presents new challenges (Khatri and Perrone 2020).

The current opioid crisis did not happen quickly; in fact, it has been described as occurring in three phases. The first phase began in the late 1990s with an increase in the number of prescription opioids. This led to overdose deaths that were attributable to natural and semi-synthetic opioids, such as methadone (Kolodyny et al. 2015; CDC 2017). The second phase began in 2010 in which heroin took the lead as the principal cause of overdose deaths. The most recent, third wave, began in 2013 in which highly potent synthetic opioids, such as fentanyl and its analogs became the main cause of mortality (Kolodyny et al. 2015; CDC 2017). The entry of fentanyl and its analogs into the clandestine market has changed the dynamics of the opioid market in the U.S. The synthetic opioids, such as fentanyl, are several orders of magnitude more potent than morphine, easily smuggled, and frequently and inconsistently mixed with lower quality drugs increasing the probability of overdosing. According to the National Forensic Laboratory Information System of the U.S. Drug Enforcement Administration (DEA), fentanyl accounted for one-third of the illicit opioids seized in 2017 (UNODC 2019c) and has become a global problem (UNODC 2019b).

### The Pathophysiology of Opioid Abuse

The effects of opioid abuse on the central nervous system (CNS) have been extensively examined. Immediate effects of opioids result in decreased levels of consciousness, sedation (Collett 1998; Thompson 2000; Indelicato and Portenoy 2002), drowsiness, and sleep disturbances (Moore and Dimsdale 2002; Bourne and Mills 2004; Qureshi and Lee-Chiong 2004). While acute opioid exposure can impair cognition in healthy subjects (Lawlor 2002; Ersek et al. 2004), enduring cognitive and psychomotor deficits occur with chronic opioid use (Sjogren et al. 2000; Dublin et al. 2015; Roberts et al. 2018; Wollman et al. 2019; Serafini et al. 2020), including altered pain perception (opioid-induced hyperalgesia), dysregulated reward/saliency processing, hyperkatifeia, and epigenetic changes, which can persist years following abstinence (Ersche et al. 2006; Browne et al. 2020). The behavioral changes seen with long-term opioid use are accompanied by lasting structural and epigenetic (e.g., altered DNA methylation and expression of non-coding RNAs) alterations in brain regions implicated in mood, reward, and motivation (Upadhyay et al. 2010; Dublin et al. 2015; Volkow and Morales 2015; Koob and Volkow 2016; Serafini et al. 2020).

Up to 90% of post-mortem tissues sampled from opiate abusers display brain edema (Buttner 2011), astrogliosis and microgliosis especially in the hippocampus (Oehmichen et al. 1996), white matter, and subcortical regions at autopsy (Tomlinson et al. 1999; Anthony et al. 2005; Buttner et al. 2006; Buttner and Weis 2006). The reactive gliosis is accompanied by increases in proinflammatory cytokines and inflammatory mediators, including TNF-α, IL-1β, and nitric oxide synthase (NOS) (Dyuizen and Lamash 2009). Opiates especially drive the enhanced activation of heme-oxygenase, NOS, and cyclic GMP-dependent-protein kinase (Liang and Clark 2004) and production of reactive nitrogen species (RNS) such as peroxynitrite (Salvemini 2009), and resultant nitrosative damage (Zou et al. 2011). Nitrosative damage is an important endpoint for opiate exposure (Pasternak et al. 1995; Liang and Clark 2004; Salvemini 2009) and key site of convergence for the oxidative stress accompanying HIV protein exposure (Hauser and Knapp 2014; McLane et al. 2018).

For delayed heroin overdose death after a survival period of 5 h or more, studies report neurovascular disorders, hypoxic ischemic leukoencephalopathy, and region-specific atrophy with neuronal losses that can include the hippocampal formation, the cerebellar Purkinje cell layer and olivary nucleus (Protass 1971; Ginsberg et al. 1976; Gosztonyi et al. 1993), as well as other areas (Buttner 2011; Cadet et al. 2014). Loss of neurons and synaptic connections is supported by postmortem reports of smaller mean relative volumes in various brain regions in individuals with OUD, including cortical areas (Danos et al. 1998; Pezawas et al. 1998), the basal ganglia (Muller et al. 2015, 2019), prefrontal cortex (Cadet et al. 2014), and hypothalamus (Muller et al. 2018). Interestingly, leukoencephalopathy, atrophy (Cadet et al. 2014), and increased hyperphosphorylated tau-containing neurofibrillary tangles are reported with chronic opiate abuse compared to age-matched controls (Ramage et al. 2005; Anthony et al. 2010; Kovacs et al. 2015). Glycogen synthase kinase 3 α or β (GSK-3α/β; the pan antibody used in this study does not discern α from β isoforms) and/or cyclin-dependent kinase-5 (Cdk-5) are increased in the frontal and temporal cortices, the locus coeruleus, and the hippocampus, respectively, and correlate with microgliosis (Anthony et al. 2010). Further, more prolonged use increases the risk of accelerated age-related and even Alzheimer’s-like pathological changes (Ramage et al. 2005; Anthony et al. 2010; Kovacs et al. 2015) and cognitive impairment (Gruber et al. 2007).

Moreover, heroin use is associated with symmetric T2 and fluid-attenuated inversion recovery (FLAIR) hyperintense white matter lesions of the CNS using magnetic resonance imaging (MRI), which coincide with increased microgliosis and inflammation at the same sites (Upadhyay et al. 2010; Bora et al. 2012; Qiu et al. 2013; Alaee et al. 2014; Li et al. 2016; Shrot et al. 2017). Although a few studies have started to examine opiate-HIV interactions in white matter (see below), we predict that the interactive effects on myelin dysregulation will significantly worsen CNS outcomes.

Preclinical studies indicate opioid-induced neuroimmune signaling alter the saliency of opioid reward and physical dependence (Narita et al. 2006; Hutchinson et al. 2008, 2009). Direct injections of astrocyte-conditioned medium containing cytokines into the nucleus accumbens (NAc) increase morphine conditioned place preference (Narita et al. 2006). Drugs reported to selectively attenuate glial inflammation block morphine conditioned place preference and attenuate symptoms of opioid withdrawal (Narita et al. 2006; Hutchinson et al. 2009; Liu et al. 2010). μ (MOR), δ (DOR), and κ (KOR) opioid receptors are expressed by subsets of astrocytes and microglia (Stiene-Martin and Hauser 1991; Eriksson et al. 1992; Stiene-Martin et al. 1993; Ruzicka et al. 1995; Gurwell et al. 1993; Hauser et al. 1996; Turchan-Cholewo et al. 2008; Maduna et al. 2018) and are involved in opioid tolerance and dependence to varying degrees (Kieffer and Gaveriaux-Ruff 2002; Berger and Whistler 2010; Morgan and Christie 2011). Despite some reports of morphine triggering immune activation via Toll-like receptor 4 (TLR4) (Terashvili et al. 2008; Hutchinson et al. 2010; Coller and Hutchinson 2012; Hutchinson et al. 2012; Theberge et al. 2013; Lacagnina et al. 2017) by binding to a myeloid differentiation protein-2 intermediary (Wang et al. 2012), this is contrary to the typical actions of opiates, which by themselves (and in the absence of a priming event such as HIV co-exposure) tend to suppress immune function (Eisenstein 2019). A vast majority of the immune, antinociceptive, and other physiological effects of opioids are mediated by opioid receptors per se and not TLR4 (Hu et al. 2011; Fukagawa et al. 2013; Stevens et al. 2013; Mattioli et al. 2014; Eisenstein 2019).

Overall, the findings indicate that immune signaling plays a critical role in the pathophysiology of OUD and associated physical dependence. How opioids effect neuroHIV, as well as how opioid abuse and dependence are altered by neuroHIV or whether opioid-HIV interactions result in a unique disease state are discussed.

## HIV Neuropathology in the Context of Opioid Use Disorder – Clinical and Preclinical Evidence

### Preclinical and Clinical Findings—a Complicated Picture

People infected with HIV (PWH) with OUD have an increased incidence of neuroHIV and CNS complications (Bell et al. 1998; Nath et al. 1999, 2000a, 2002; Anthony et al. 2008; Meyer et al. 2013; Smith et al. 2014). Injection drug use increases the probability of contracting HIV (Nath et al. 1999) and opioid drugs intrinsically alter the pathogenesis of HIV. PWH who develop intractable pain syndromes related to peripheral neuropathies often receive opioid drugs for treatment (Mirsattari et al. 1999; Denis et al. 2019). PWH who misuse opioids are more likely to undertake risky sexual behavior and are less likely to adhere to combined antiretroviral (ARV) therapy (cART) regimens (Lemons et al. 2019). Opioid receptors are widely expressed on immune cells and opioids can modulate immune function (Donahoe and Falek 1988; Plotnikoff 1988; Rouveix 1992; Adler et al. 1993; Carr and Serou 1995; Carr et al. 1996; Sheng et al. 1997; Banerjee et al. 2011; Purohit et al. 2012), which typically (but not always) result in immune suppression (Wybran et al. 1979; McDonough et al. 1980, 1981; Donahoe and Falek 1988; Donahoe et al. 1991; Falek et al. 1991; Novick et al. 1991; Chao et al. 1996a; Peterson et al. 1998; Rogers and Peterson 2003; Stein et al. 2003; Roy et al. 2006; Rittner et al. 2008). The “opiate cofactor hypothesis” proposes opioids contribute directly to the pathogenesis of acquired immune deficiency syndrome (AIDS) (Donahoe and Vlahov 1998), in part, because MOR activation can increase HIV replication in immune cells (Peterson et al. 1990, 1992, 1993, 1999; Ho et al. 2003). Furthermore, MOR and HIV co-receptors, including both CCR5 (El-Hage et al. 2013; Yuan et al. 2013; Arnatt et al. 2016) and CXCR4 (Pitcher et al. 2014) can interact via convergent downstream signaling and perhaps via direct molecular interactions (Rogers et al. 2000; Rogers and Peterson 2003; Steele et al. 2003; Chen et al. 2004; Song et al. 2011; Arnatt et al. 2016). MOR-CCR5 or CXCR4 interactions are highly contextual and can promote (Guo et al. 2002; Steele et al. 2003) or inhibit (Strazza et al. 2014) HIV expression, depending on the nature and duration of exposure (see Fig. 9; Berman et al. 2006) and cell type involved (Kim et al. 2018). Depending on the outcome measure, Tat expression reduces morphine’s efficacy and potency (Fitting et al. 2012, 2016; Hahn et al. 2016). Antagonizing CCR5 with maraviroc reinstates morphine potency in an antinociceptive assay and restores physical dependence in Tat exposed, morphine-tolerant mice (Gonek et al. 2018).

Epidemiological studies suggest OUD can increase AIDS progression (Donahoe and Vlahov 1998; Dronda et al. 2004; Meijerink et al. 2014, 2015). In the pre-cART era, opiate abuse was found to exacerbate HIV encephalitis (HIVE) (Bell et al. 1998, 2002). In Indonesian injection heroin abusers who lacked access to cART, CD4 counts (a measure of HIV progression) were reduced compared to PWH not using heroin (Meijerink et al. 2014). However, with the introduction of cART, the clinical picture has significantly changed with a 50% decline in the rate of death from AIDS, reduced incidence of opportunistic infections and HIVE, and a 40–50% decrease in the incidence of HIV-associated dementia (HAD), the most severe form of HIV-associated neurocognitive disorders (HAND) (Maschke et al. 2000; McArthur et al. 2010; Saylor et al. 2016). Nevertheless, chronic opiate exposure (which almost always is confounded by the use of other illicit and legal drugs) in PWH can worsen neuroHIV (Anthony et al. 2005; Bell et al. 2006; Anthony et al. 2008) and cognitive impairment (Rodriguez Salgado et al. 2006; Martin-Thormeyer and Paul 2009; Byrd et al. 2011; Smith et al. 2014; Martin et al. 2018; Rubin et al. 2018) despite cART, even though some studies fail to show that opioids worsen neuroHIV (Royal et al. 1991; Applebaum et al. 2010) or HAND (Martin et al. 2019). Opiate exposure in cART-treated PWH worsens CD4 counts and viral loads (Ryan et al. 2004), neuropathology (including increased tauopathy; Smith et al. 2014), CNS inflammation (Anthony et al. 2005, 2008; Smith et al. 2014), and neurocognition (Applebaum et al. 2009; Byrd et al. 2011; Meyer et al. 2013) including deficits in memory and working memory (Byrd et al. 2011). Table 1 gives an overview on reported interactive effects of HIV and opioids in some of the clinical and preclinical CNS studies referenced in this review.

**Table 1:** Clinical and preclinical findings

Major effects	HIV pathogen^a^	ARV	Opioids	Outcome	Model system	Citation(s)
Clinical findings (human)						
HIV progression and/or ARV adherence	HIV	cART	• SUD • Prescription opioids for pain	• ↑ Viral load with SUD • ↓ ARV adherence • ↑ Frequency of prescription drugs with pain + SUD	Human	(Denis et al. 2019)
	HIV	cART	OUD	• ↓ Lasting viral suppression • ↓ Adherence to cART for 3 years	Human	(Lemons et al. 2019)
	HIV	ARV naive	Injection drug use	↓ CD4 counts	Human	(Meijerink et al. 2014)
HIV encephalitis (HIVE) HIV infection CNS	HIV	ZDV	Former drug use (+ OST)	• ↑ Multinucleated giant cells • ↑ HIV p24	Human, postmortem brain	(Bell et al. 1998)
Microglial activation	HIV	• ARV • ZDV	OUD	↑ CD68 microglial activation only in non-OUD HIV+ PWH	Human, postmortem brain	(Smith et al. 2014)
	HIV	• ARV • ZDV, other monotherapies	Injection drug use (+ OST)	↑ Microglial activation	Human	(Bell et al. 2002)
	HIV	No info	Drug use	• ↑ MHC class II • ↑ CD68	Human, postmortem brain	(Anthony et al. 2005)
	HIV	No info	OUD (44% methadone, 36% other opiates)	• ↓ CD68, HLA-D in HIV and HIVE with OUD • No effect of IDU on CD68	Human, postmortem brain	(Byrd et al. 2012)
Plasma cytokines	HIV	cART	OUD (codeine, fentanyl, morphine)	↑ sTNF-R2, not sCD14, TNF-α, sTNF-R1, in plasma	Human	(Ryan et al. 2004)
	HIV	ARV naive	Reported heroin use	• ↓ MIP-1α, MIP-1β, MCP-2 in blood after stimulation with LPS • ↑ CCR5 expression in CD4 cells	Human	(Meijerink et al. 2015)
HIVE	HIV	No info	OUD	• ↑ Parenchymal inflammatory infiltrates • ↑ HIV PCR amplification products	Human, postmortem brain	(Gosztonyi et al. 1993)
Aberrant immune responses	HIV	No info	SUD (opioids, alcohol, marijuana, cocaine) (+ OST)	• ↑ Autoantibodies and delayed hypersensitivity to neural antigens OUD only • No HIV effect/interaction	Human	(Jankovic et al. 1991)
Learning-memory	HIV	50-70% on cART	Heroin, crack/cocaine	• ↓ Total learning; ↓ Learning slope • ↓ Delayed recall	Human, female	(Meyer et al. 2013)
	HIV	cART	Reported heroin use	• ↓ Recall memory • ↓ Working memory	Human	(Byrd et al. 2011)
	HIV	No info	SUD (opioids, alcohol, marijuana, cocaine)	• ↓ Complex figure copy • ↓ Delayed recall	Human	(Concha et al. 1997)
Neuropsychological performance		cART	OST (methadone)	No effect of OST	Human	(Applebaum et al. 2010)
Cognitive function	HIV	cART	OUD	• ↓ Cognitive performance with anticholinergics, but not opioids, anxiolytics, or anticonvulsants	Human	(Rubin et al. 2018)
Memory Cognitive function	HIV	cART	SUD (alcohol, cocaine, heroin)	• ↓ Working memory in HIV+ • ↓ Spatial and verbal response times in women, irrespective of HIV status • ↑ Response time with cocaine use	Human	(Martin et al. 2018)
Visual and cognitive function	HIV	No info	OUD (+ OST, methadone)	• ↑ Pattern-shift visual evoked potential delay with methadone • No HIV effect/interaction	Human	(Bauer 1998)
Transmission risk	HIV	No info	OST	↓ Frequency of injection drug use	Human	(Kwiatkowski and Booth 2001)
	HIV	cART	OST	• ↓ Frequency of heroin injection • ↑ On ARV	Human	(Pettes et al. 2010)
Motor and visual function	HIV	No info	OST	• ↓ Digital Finger-Tapping test • ↓ Visual motor pursuit	Human	(Silberstein et al. 1993)
ARV adherence	HIV	cART	OST	• ↑ ARV adherence in PWH with OST vs. OUD	Human	(Mazhnaya et al. 2018)
*PENK* expression	HIV	Pre- and post-cART	SUD	• ↓ *PENK* in HIVE vs. HIV− • ↓ *DRD2L* HIV+ vs. HIVE & HIV− • ↓ *DRD2L* correlates with ↑ cognitive performance	Human, post mortem brain	(Gelman et al. 2012)
*OPRM1* polymorphisms, splice variants	HIV	No info	SUD	C17T MOR polymorphism correlates with ↑ risk of cocaine, alcohol & tobacco (but not opiate) use	Human	(Crystal et al. 2012)
	HIV	cART	No	Some *OPRM1* polymorphisms may alter HIV severity / response to ARV	Human	(Proudnikov et al. 2012)
	HIV	No info	MOR-1K expression	• ↑ MOR-1K in HIVE • ↑ CCL2, CCL6, CCL5, but not CXCR4, CCR5 or CD4 receptor in HIVE	Human, postmortem brain	(Dever et al. 2014)
*OPRK* and *PDYN* polymorphisms	HIV	cART	No	Some *OPRK and PDYN* polymorphisms may alter HIV severity / response to ARV	Human	(Proudnikov et al. 2013)
Sensory Neuropathy	HIV	cART	SUD	HIV sensory neuropathy- regardless of SUD (trends, not significant)	Human	(Robinson-Papp et al. 2010)
Preclinical in vivo findings (animal)						
HIV entry into the brain	Mixture of SIV_17-EFr_, SHIV_KU_1B,_ SHIV_89.6P_	No	Morphine (5 mg/kg i.m., b.i.d., ≤ 56 weeks)	• ↑ CSF viral load • ↑ Viral migration through BBB for SHIV_KU_	Rhesus macaques	(Kumar et al. 2006)
	SIV_macR71/17E_	No	Morphine (3 mg/kg i.m., q.i.d.)	• ↑ CD4+ and CD8+ T cells • ↑ CSF viral load • ↑ Infiltration of MDMs into the brain	Rhesus macaques	(Bokhari et al. 2011).
Viral load and HIV progression	Mixture of SIV_17-EFr_, SHIV_KU _1B_, SHIV_89.6P_	No	Morphine (5 mg/kg, i.m., t.i.d., 20 weeks)	• ↑ Viral load; ↓ CD4 counts • ↑ ROS with morphine + SIV	Rhesus macaques	(Perez-Casanova et al. 2007; Perez-Casanova et al. 2008)
SIV gene mutation/evolution*tat*	Mixture of SIV_17-EFr_, SHIV_KU _1B_, SHIV_89.6P_	No	Morphine (5 mg/kg, i.m., t.i.d., 20–56 weeks)	• ↑ Viral load; ↓ CD4 counts • *tat* evolution—inverse correlation with SIV progression • ↓ *tat* diversity with morphine	Rhesus macaques	(Noel and Kumar 2006; Noel et al. 2006b)
*nef*				• ↑ Viral load; ↓ CD4 counts • ↓ *nef* evolution; no correlation with SIV progression ± morphine		(Noel et al. 2006a)
*env*				• ↑ Viral load; ↓ CD4 counts • ↑ *env* evolution (V4 region) correlates with SIV progression + morphine • ↑ *env* evolution in CSF with morphine		(Rivera-Amill et al. 2007, 2010b)
*vpr*				• ↓ *vpr* evolution and/or Vpr R50G mutation—inverse correlation with SIV progression/mortality • ↓ *vpr* evolution with morphine		(Noel and Kumar 2007; Rivera et al. 2013)
Neuronal injury, survival, oxidative stress	gp120 HIV-1_LAV_	No	Morphine (25 mg pellet, 5–7 days)	• ↑ ROS during withdrawal • ↓ PSD95 during chronic and withdrawal • ↑ Sphingomyelin • ↓ Ceramide	Mouse, gp120 tg^b^	(Bandaru et al. 2011)
	HIV	No	Morphine (37.5 mg s.c, 5 days)	↓ neuron survival HIV tg + morphine	Rat, HIV-1 tg, female	(Guo et al. 2012)
	SIV HIV Tat	No	Morphine (3 mg/kg i.m., q.i.d., 3 weeks)	• ↑ miR-29b, ↓ PDGF-B mRNA, ↑ PDGF-BB with morphine and SIV • ↓ PDGF-B, ↓ neuron survival with CM from morphine-treated astrocytes	Rhesus macaques; Rat^b^, primary neurons, astrocytes	(Hu et al. 2012)
Synaptic transmission	Tat_1–86_	No	Morphine ex vivo (1 μM) to the bath	↓ mIPSC frequency	Mouse, male and female, PFC slices, ex vivo	(Xu and Fitting 2016)
	SIV_macR71/17E_ Tat	No info	• Morphine (escalating doses of 1–3 mg/kg i.m., q.i.d., 12 months) • Morphine in vitro	• SIV ↑ Synaptic protein HSPA5 • Tat ↑ HSPA5 mRNA (in vitro)	Rhesus macaques; Human, SH-SY5Y neuroblastoma cells in vitro	(Pendyala et al. 2015)
White matter effects	SIV_macR71/17E_	No	Morphine (3 mg/kg i.m., q.i.d., ≤ 59 weeks)	• ↑ Focal, demyelinating lesions • ↑ Macrophages in areas of myelin loss	Rhesus macaques	(Marcario et al. 2008),
CNS metabolites	SIV_smm9_	No info	Morphine (escalating doses of 1–3 mg/kg i.m., q.i.d., ≤ 4 years)	• ↑ Survival time • ↑ Creatine in white matter (SIV + morphine only) • ↑ Myo-inositol in putamen	Rhesus macaques	(Cloak et al. 2011)
Neuroinflammation	Tat_1–86_	No	Morphine (10 mg/kg i.p., b.i.d., 5 days)	↑ Iba1+ 3-NT+ microglia	Mouse, Tat tg, males	(Zou et al. 2011)
Chemokines	Tat_1–72_ (25 μg intrastriatal injection)	No	Morphine (25 mg pellet, 5 days)	• ↑ CCL2 in astrocytes is regulated by CCR5 • ↑ CCL2 in macrophages/microglia • CCL2-knockout blocks morphine + Tat-induced glial reactivity	Mouse	(El-Hage et al. 2008a)
Cytokines, Chemokines	HIV Tat (10 μg/kg i.v.)	No	Morphine (25, 75 mg pellet, 6 days)	• Morphine ↑ death in Tat + bacterial infection • ↑ TNFα, IL-6, CCL2, • ↑ TLR2, TLR4, TLR9	Mouse, male, in vivo; microglia in vitro	(Dutta et al. 2012)
MOR expression	HIV-1_IIIB_ gp120 (X4)	No	MOR	↑ MOR mRNA	Rats, HIV-1 tg males	(Chang et al. 2007)
MOR-coupling efficacy to G proteins	Tat_1–86_	No	• Morphine (acute, 10 mg/kg i.p.) • Morphine, DAMGO (ex vivo)	↓ [^35^S]GTPγS binding in NAc Shell, CPu, amygdala, PFC, but not hippocampus, with morphine in Tat mice	Mouse, Tat tg, males	(Hahn et al. 2016)
Neuroinflammation; morphine tolerance (antinociception), physical withdrawal, reward	Tat_1–86_	No	Morphine (75 mg pellet, 5 days)	• ↑ Tolerance (↓ anti-nociceptive potency and ↓ withdrawal symptoms) • ↑ CPP and cytokines (24 h after withdrawal) • Above effects reduced by CCR5 blockade	Mouse, Tat tg, males	(Gonek et al. 2018)
Neuropathy	gp120 (0.2 μg), q.d. intrathecally	No	Morphine (3 μg, intrathecally, b.i.d., 5 days)	• ↑ Mechanic allodynia • ↑ Brd4 mRNA	Rat, males, gp120	(Takahashi et al. 2018)
Morphine efficacy, potency	Tat_1–86_	No	Morphine (acute, 2–8 mg/kg s.c.)	↓ Antinociceptive potency and efficacy (tail flick)	Mouse, Tat tg, males	(Fitting et al. 2012)
Morphine tolerance, physical dependence	Tat_1–86_	No	Morphine (75 mg pellet, 4 days)	• ↑ Antinociceptive tolerance • ↓ Physical dependence	Mouse, Tat tg, males	(Fitting et al. 2016)
Locomotor function	Tat_1–86_	No	Oxycodone (0–10 mg/kg, i.p., 15 min prior behavioral assay)	↑ Locomotor activity, center entries (open field)	Mouse, Tat tg, females	(Salahuddin et al. 2020)
	SIV_macR71/17E_	No	Morphine (escalating doses of 1–2.5 mg/kg i.m., q.i.d., 59 weeks)	↓ Motor skill	Rhesus macaques	(Marcario et al. 2016)
	Tat_1–86_	No	Oxycodone (acute, 0.1–10 mg/kg, i.p.)	↑ Psychomotor effects	Mouse, Tat tg, females	(Paris et al. 2020)
BBB integrity	Tat	No	Morphine (25 mg pellet, 5 days)	↑ Dextran extravasation across the blood-brain barrier	Mouse, Tat tg females	(Leibrand et al. 2019)
Immune cell trafficking into CNS	Tat	No	Morphine	• ↑ Infiltration of monocytes and T cells into *S. pneumoniae*-infected CNS with morphine • ↑ T cell CXCR4 and CCR5 expression with morphine	Mouse, CNS infection (*S. pneumoniae)*, males	(Dutta and Roy 2015)
ARV accumulation	Tat	DTG ABC 3TC	Morphine (2 mg/day, s.c.. osmotic pump, 5 days)	↓ Dolutegravir and abacavir, but no change in lamivudine in brains of morphine-treated animals	Mouse, Tat tg females	(Leibrand et al. 2019)
Circadian rhythms	Tat_1–86_	No	Morphine (25 mg pellet, last 5 days)	↓ Total wheel-running activity	Mouse, Tat tg, males	(Duncan et al. 2008)

^a^assumed Clade B, unless noted otherwise; ^b^ sex not reported; ^c^ authors reported a trend that was not significant

*ABC*, abacavir; *ARV*, antiretroviral(s); *BBB*, blood-brain barrier; *b.i.d.*, twice a day; *Brd4*, Bromodomain-containing protein 4; *CPu*, caudate-putamen; *CNS*, central nervous system; *CPP*, conditioned place preference; *CM*, conditioned medium; *CSF*, cerebrospinal fluid; *DAMGO* [D-Ala^2^, N-MePhe^4^, Gly-ol]-enkephalin; *DRD2L*, type 2 dopamine receptor; *DTG*, dolutegravir; *HIVE*, HIV encephalitis (typically seen pre-cART); *HSPA5*, heat shock 70-kDa protein A 5; *IDU*, injection drug use; *i.m*., intramuscularly; *i.p*., intraperitoneal; *Iba1*, ionized calcium-binding adapter molecule 1; *3TC*, lamivudine; *MHC class II*, major histocompatibility class II; *mIPSC*, miniature inhibitory postsynaptic currents; *MOR*, μ-opioid receptor; *No info*, information not provided or uncertain; *OST*, opioid substitution therapy; *OUD*, opioid use disorder; *PFC*, prefrontal cortex; *PENK*, preproenkephalin; *q.d*., once a day; *q.i.d.*, four times a day; *ROS*, reactive oxygen species; *s.c.*, subcutaneous; *SUD*, substance use disorder; *tg*, transgenic; *t.i.d*., three times a day; *ZDV*, zidovudine

For practicality, Tables 1 and 2 are limited to key studies in the CNS with emphasis on neuropathological or neuroimmune rather than psychosocial outcomes. With deference toward the excellent studies we excluded: (1) on opioid and HIV effects on peripheral blood mononuclear cells (PBMCs), or on isolated lymphocytes and monocytes, not directly related to the central nervous system or BBB; (2) on HIV or opioid and ARV interactions in the peripheral nervous system; and (3) studies not directly examining opioid-HIV interactions (irrespective of whether a positive or negative interaction was found)

Although translational, “bench-to-bedside”, research is important, reverse-translational approaches and multiple preclinical models are essential to better understand complex disease and improve established therapies (Singer 2019). Evidence suggests that HIV compartmentalizes within the CNS early during the course of the infection establishing a separate reservoir harboring “intact proviral” HIV (Churchill et al. 2016; Bruner et al. 2019) within resident neural cell populations (Bednar et al. 2015; Sturdevant et al. 2015; Veenhuis et al. 2019) and perivascular macrophages (Fischer-Smith et al. 2001; Burdo et al. 2013; Rappaport and Volsky 2015). Preclinical studies assessing opioid interactions with HIV or viral proteins permit mechanistic understanding of how particular CNS cell types, including neurons, astroglia, and microglia are affected and contribute to accentuating effects of opiates on neuroHIV, which are discussed in detail below.

### Cellular and Molecular Interactions in Astroglia, Microglia, and Neurons

Prior reviews have outlined how opiate drugs likely exacerbate neuroHIV pathology in neurons and glia (Hauser et al. 2005; Dutta and Roy 2012; Hauser et al. 2012; Reddy et al. 2012; Hauser and Knapp 2014; Liu et al. 2016a; Murphy et al. 2019) including in the enteric nervous system (Galligan 2015; Meng et al. 2015). Opioid-HIV pathophysiological interactions are complex and differ depending on the timing and duration of co-exposure, the pharmacology of the opioid drug involved, the cell types and brain regions targeted, host and viral genetics, and are highly contextual (Hauser and Knapp 2014, 2018). A summary of the cellular and molecular interactions in various CNS cell types is also reviewed in detail in Table 2.

**Table 2:** Cellular and molecular interactions (in vitro)

Major effects	HIV pathogen^a^	ARV	Opioids	Outcome	Model system (in vitro)	Citation(s)
Mixed-Glia						
HIV expression	HIV	No	• Dynorphin • U50,488 (KOR agonists)	• ↑ HIV-1 expression, • Dynorphin (KOR agonist) ↑ TNF-α, IL-6 mRNA and protein	Human fetal neural cells, HIV-infected promonocyte (U1) line	(Chao et al. 1995)
	HIV_SF162_	No	• U50,488 • U69,593 • Dynorphin_1–17_; (KOR agonists) • Morphine	• KOR agonists ± TNF-α differentially ↓ HIV p24	Human, primary mixed neurons and glia	(Chao et al. 1998a)
Chemokines	Tat_1–86_	No	Morphine	• ↑ CCL5, CCL2 • ↑ [Ca^2+^]_i_ (Beclin1 dependent) • ↓ Autophagy	Mouse, primary mixed glia	(Lapierre et al. 2018)
	HIV_SF162_ (R5)	No	Morphine	• ↑ HIV-1 Tat-induced LTR expression • ↑ CCR5 expression (inhibited by bivalent ligand in astrocytes) • ↑ IL-6 • ↑ CCL5	Human, primary mixed glia	(El-Hage et al. 2013)
Glial restricted precursors: survival & MOR, DOR, KOR expression	Tat_1–72_	No	Morphine (acting via DOR and/or KOR)	• ↑ Caspase-3 activation & ↑ cell death by Tat or morphine via DOR, KOR • No opioid-Tat interactions	Mouse, primary glial precursors	(Buch et al. 2007)
MOR expression in NPCs; NPC survival and developmental fate	Tat_1–72_	No	Morphine	• MOR expressed by subsets of NPCs • ↑ Astrocyte and immature glial death	Mouse, primary mixed glia	(Khurdayan et al. 2004)
MOR and CCR5 interactions	Tat_1–86_ (from HIV_IIIB_)	No	Morphine	• ↓ Neuronal survival via CCR5 activation in glia (rescued by BDNF treatment)	Mouse, primary neurons and glia	(Kim et al. 2018)
HIV infectivity MOR-CCR5 dimerization	HIV_SF162_ (R5)	No	Morphine CCR5-MOR bivalent ligand 1b	• MOR-CCR5 bivalent ligand blocks HIV infection in astroglia, but not microglia, with morphine • MOR-CCR5 bivalent ligand blocks the fusion of HIV gp160 and CCR5-CD4-expressing HEK cells	Human, primary astrocytes and microglia; HEK-293T cells	(Yuan et al. 2013; Arnatt et al. 2016)
HIV expression and maturational fate of neurons and astroglia	HIV_BaL_ (R5)	No	Morphine	• ↑ HIV p24 and ↑ Tat mRNA levels with morphine after 21 days • ↓ Proliferation of neural progenitors; ↑ astroglial and ↑ neuronal differentiation	Human, neural progenitors	(Balinang et al. 2017)
Astrocytes						
HIV expression	HIV_SF162_ (R5)	No	Morphine	• ↑ HIV p24 • ↑ CCL2	Human, primary astrocytes	(Rodriguez et al. 2017)
Toll-like receptor (TLR) expression/function	• Tat_1–72_ • gp120	No	Morphine	• ↑ TLR2 with Tat, Tat + morphine, gp120 • ↓ TLR9 with Tat, morphine, gp120	Mouse, primary astrocytes	(El-Hage et al. 2011a)
Chemokines	Tat_1–72_	No	Morphine	• ↑ CCL5, CCL2 • ↑ IL-6 • ↑ [Ca^2+^]_i_	Mouse, primary astrocytes	(El-Hage et al. 2005)
	Tat_1–72_	No	Morphine	• ↑ CCL2 • ↑ CCL5 • ↑ Microglial migration	Mouse, primary astrocytes	(El-Hage et al. 2006a)
	Tat_1–72_	No	Morphine	• ↑ CCL2, ↑ IL-6, ↑ TNF-α • ↑ [Ca^2+^]_i_ • ↑ NF-κB trafficking and transcription • No interaction / acceleration with morphine	Mouse, primary astrocytes	(El-Hage et al. 2008b)
	Tat	No	• U50,488 (KOR agonist) • Nor-BNI (KOR antagonist)	• U50,488 ↓ CCL2 • U50,488 ↓ NF-κB	Human, primary astrocytes	(Sheng et al. 2003)
	N/A	No	Morphine	• ↑ CCR5, CCR3, CXCR2 • ↓ IL-8, CCL4	Human, astrocytoma U87 cell line, primary astrocytes	(Mahajan et al. 2002)
	• Tat_1–86_ • gp120_IIIB_	No	Morphine	Regional differences in cytokine and ROS production —differed for each insult	Mouse, primary astrocytes	(Fitting et al. 2010a)
Oxidative stress / damage	Tat_1–72_	No	• DPDPE • SNC-80 (DOR agonists)	DOR agonists ↓ Tat-induced oxidative stress	Human derived brain cell line (SK-N-SH)	(Wallace et al. 2006)
Inflammation, maturation /plasticity	• Tat_86_ • Tat_101_	No	Morphine	↓ β-catenin signaling and variably decreases *TrkB*, *BDNF*, and *NLRP1* mRNA in fetal astrocytes ^b^	Human, U87MG and fetal astrocytes	(Chen et al. 2020)
Microglia						
HIV replication	HIV_SF162_ (R5)	No	• Endomorphin-1 • Endomorphin-2 (MOR agonists)	• ↑ HIV p24 with endomorphin-1, but not endomorphin-2 • Endomorphin-1 acts via MOR, but not DOR / KOR	Human, primary microglia	(Peterson et al. 1999)
	HIV_SF162_ (R5)	No	Morphine	↑ HIV p24	Human, primary microglia	(El-Hage et al. 2014)
	HIV_SF162_ (R5)	No	• U50,488; U69,593 (KOR agonists) • Dynorphin A_l-13_	↓ HIV p24	Human, primary microglia	(Chao et al. 1996b)
	• HIV_JR-FL_ (R5) • gp120	No	β-endorphin	• ↑ HIV expression • ↑ HIV p24 (14-day post infection) • gp120 ↑ IL-1, TNF, IL-6	Human, fetal microglia	(Sundar et al. 1995)
	HIV_SF162_	No	• 8-CAC, U50,488 (KOR agonists) • Cocaine	• KOR agonist ↓ p24; blocked by KOR antagonists • KOR agonist negates cocaine-induced ↑ HIV	Human, fetal brain microglia	(Gekker et al. 2004)
	HIV_SF162_	No	*OPRL1* antisense Nociceptin / orphanin FQ (OPRL1 agonist)	• *OPRL1* antisense (*and sense*) ↓ p24 • Nociceptin, no effect on p24	Human, fetal brain microglia and mixed neurons/glia	(Chao et al. 1998b)
HIV expression	• HIV_SF162_ • Tat	ZDV	U50,488 (KOR agonist)	• ↓ p24 on day 14 with U50,488 • ↓ Neurotoxicity (U50,488) • ↓ Quinolinate by microglia	Human, fetal microglia and neural cells	(Chao et al. 2000)
Chemokines and Cytokines	Tat_1–72_	No	Morphine	• ↑ CCR5 • ↑ CD11b, ↑ CD40 • ↑ TNF-α, ↑ IL-6, ↑ IP-10 • ↑ iNOS	Mouse, BV-2 and primary microglia	(Bokhari et al. 2009)
MOR signaling	Tat_1–72_	No	Morphine	• ↑ MOR (intracellular) • ↑ MOR mRNA	Mouse, N9 and primary microglia	(Turchan-Cholewo et al. 2008)
Oxidative Stress	Tat_1–72_	No	Morphine	• ↑ ROS [O_2_^−^ (DHE), ↑ HO_2_^•^, H_2_O_2_ (DCF)] • ↑ Protein carbonyls	Mouse, N9 and primary microglia	(Turchan-Cholewo et al. 2009)
Glutamate release	Tat_1–72_	No	Morphine	↑ Glutamate release via ↑ x_c_^−^ cystine-glutamate antiporter expression/function	Mouse, primary microglia	(Gupta et al. 2010)
Neurons						
HIV expression	HIV	No	Morphine	↑ HIV expression	Human derived, SH-SY5Y neuroblastoma cells	(Squinto et al. 1990)
Homeostasis and Injury	Tat_1–86_	No	Morphine	• ↑ [Ca^2+^]_i,_ • ↑ [Na^+^]_i_ • ↓ Δ_Ψm_ (mitochondrial) instability • ↑ Dendritic degeneration	Mouse, primary neurons	(Fitting et al. 2014a)
Mitochondrial inner membrane potential and ROS	• Tat_1–86_, Tat_1–72_ • gp120	No	Morphine	↑ Δ_Ψm_ instability and oxidative stress ↑ with Tat + morphine, ↑ neuroprotection with allopregnanolone	Human, primary neurons ; mouse, striatal medium spiny neurons; mouse, striatal medium spiny neurons, SH-SY5Y neuroblastoma cells	(Turchan-Cholewo et al. 2006; Paris et al. 2020)
Neuronal survival	Tat_1–86_	No	Morphine	• ↓ Neuronal survival from Tat + morphine and ↓ glial CX_3_CL1 rescued by CX_3_CL • CX_3_CL1 (fractalkine) regulates microglial motility	Mouse, primary neurons and mixed glia	(Suzuki et al. 2011)
	Tat_1–86_	No	Morphine	• ↓ Proliferation • ↑ ERK1/2 activation • ↑ p53 and p21 • ↓ Cyclin D1 and Akt levels	Human, neuronal precursors	(Malik et al. 2014)
	Tat_1–72_, Tat_1–86_	No	Morphine	• ↓ Neuronal survival • ↑ Neuronal survival with ibudilast (AV411) (inhibiting glial NF-κB blocks Tat ± morphine neurotoxicity)	Mouse, primary neurons and mixed glia	(Gurwell et al. 2001; El-Hage et al. 2014)
White matter/oligodendroglial pathology						
Changes in OL survival and morphology	Tat_1–86_	No	Morphine (25 mg pellet, 7 days); morphine (in vitro)	• ↑ Degeneration of OLs • ↑ TUNEL reactivity • ↑ Caspase-3 activation	Mouse, Tat tg; primary OLs	(Hauser et al. 2009)
Blood-brain barrier and the neurovascular unit						
BBB model integrity and function	Tat_1–86_	No	Morphine	• ↑ TNF-α • ↑ IL-8 • ↓TEER • ↑ JAM-2 expression • ↑ Monocyte transmigration with CCL5	Human, using primary BMVEC and primary astrocytes	(Mahajan et al. 2008)
ARV accumulation	Tat_1–86_	DTG FTC TFV	Morphine	• ↓ Intracellular ARV concentrations	Human, primary astrocytes	(Patel et al. 2019)
HIV-1 strain differences						
Neuronal Survival	Tat_1–86_ (clades B & C)	No	Morphine	• ↓ Neuronal survival via MOR on mixed glia • ↑ ROS in astrocytes • ↑ Iba1 and 3-NT microglia with morphine	Mouse, primary neurons and mixed glia	(Zou et al. 2011)
	• gp120_IIIB_ • gp120_MN_ (X4) • gp120_ADA_ (R5)	No	Morphine	↓ Neuronal survival in presence of glia with gp120_MN_ and transiently with gp120_IIIB_ (X4), not R5-tropic gp120, in combination with morphine	Mouse, primary neurons and mixed glia	(Podhaizer et al. 2012)
Proliferation and maturational fate of neural progenitors and oligodendroglia	• HIV_SF162_ (R5) • HIV_IIIB_ (X4)	No	Morphine	• ↓ Proliferation of immature neural and OL progenitors with Tat + morphine • ↓ NPC DNA synthesis with R5-tropic HIV + morphine • ↑ NPC DNA synthesis with X4-tropic HIV + morphine	Mouse, Tat tg; Mouse, Human, primary neural progenitors	(Hahn et al. 2012)
GABA function	• HIV_BaL_ (R5) • gp120 _(ADA, MN, and IIIB)_ • Tat_1–86_	No	Morphine	• Tat or morphine ↓ KCC2 levels via CCR5 • ↑ KCC2 prevents Tat and R5 HIV, gp120, but not X4, gp120 neurotoxicity ± morphine	Human, primary neurons, hNPCs	(Barbour et al. 2020)
Astroglial CCL5 and neuroprotection	• gp120_IIIB_ (X4) • gp120_BaL_ (R5)	No	• Morphine (10 μM) • DAMGO	• Morphine ↑ astroglial CCL5 blocking gp120_BaL_ neurotoxicity • Morphine (or CXCL12) does not block gp120_IIIB_ neurotoxicity	Rat, mixed neurons and glia; isolated neurons, astrocytes and microglia	(Avdoshina et al. 2010)

^a^assumed Clade B, unless noted otherwise, ^b^ statistical findings for some results are unclear

*ARV*, antiretroviral(s); *BMVEC*, brain vascular endothelial cells; *[Ca*^*2+*^*]*_i_ intracellular calcium concentration; *8-CAC*, 8-carboxamidocyclazocine; *DAMGO*, D-Ala^2^, N-MePhe^4^, Gly-ol]-enkephalin; *DCF*, dihydro-dichlorofluorescein; *DOR*, δ-opioid receptor; *DHE*, dihydroethidium; *DTG*, dolutegravir; *DPDPE*, [D-Pen^2^,D-Pen^5^]enkephalin; *FTC*, emtricitabine; *GABA*, γ-aminobutyric acid; *Iba1*, ionized calcium-binding adapter molecule 1; *JAM-1*, junctional adhesion molecule-1; *KCC2*, K^+^-Cl^−^ cotransporter 2; *KOR*, κ-opioid receptor; *LTR*, long terminal repeat; *Δ*_*Ψm*_, mitochondrial inner membrane potential; *MOR*, μ-opioid receptor; *[Na*^*+*^*]*_i_, intracellular sodium concentration; *nor-BNI*, nor-binaltorphimine; *NPCs*, neural progenitor cells; *OLs*, oligodendroglia; *ROS*, reactive oxygen species; *TEER*, transendothelial electrical resistance; *TFV*, tenofovir; *TUNEL*, terminal deoxynucleotidyl transferase dUTP nick end labeling; *ZDV*, zidovudine

For practicality, the table is limited to key studies in the CNS with emphasis on neuropathological or neuroimmune rather than psychosocial outcomes. With deference toward the excellent studies we excluded: (1) on opioid and HIV effects on PBMCs, or on isolated lymphocytes and monocytes, not directly related to the central nervous system or BBB; (2) on HIV or opioid and ARV interactions in the peripheral nervous system; and (3) studies not directly examining opioid-HIV interactions (irrespective of whether a positive or negative interaction was found)

#### Opioid and HIV Interactive Pathology in Astroglia

Although the extent to which astroglia display productive infection is debated (Russell et al. 2017; Ko et al. 2019), there is nevertheless considerable evidence of proviral integration in the CNS of PWH (Gorry et al. 2003; Churchill et al. 2009), infectious animal models (Eugenin et al. 2011), and/or cultured human fetal astrocytes (Tornatore et al. 1994; Liu et al. 2004; Do et al. 2014; Narasipura et al. 2014; Li et al. 2015; Nath 2015; Li et al. 2020). Integrated HIV sequences have been identified in astrocytes in HIV-infected CNS tissue by laser capture microdissection (Churchill et al. 2006). Astroglia appear to infect via non-classical, CD4-independent mechanisms, that can have the appearance of virologic synapses, adding to the debate (Liu et al. 2004; Do et al. 2014; Li et al. 2015; Nath 2015; Al-Harthi et al. 2019; Li et al. 2020).

Irrespective of whether they become infected, MOR-expressing, HIV or HIV protein-exposed astrocytes release greater amounts of inflammatory cytokines and dysfunction sufficient to harm bystander neurons upon treatment with opiates (El-Hage et al. 2005, 2008b; Zou et al. 2011; El-Hage et al. 2014). MOR-expressing subsets of glia, especially microglia and astroglia, are prominent in driving the interactive opioid and HIV neuropathogenesis (Hauser et al. 2007, 2012; Hauser and Knapp 2014; Liu et al. 2016a; Chilunda et al. 2019; Murphy et al. 2019). When MOR is deleted from glia (astrocytes and microglia), morphine no longer increases the death of Tat-exposed striatal medium spiny neurons (MSNs) (Zou et al. 2011). Conversely, if MOR is deleted from MSNs, morphine exacerbates the neurotoxic effects of Tat in MSNs (Zou et al. 2011). The proinflammatory effects of Tat alone or in combination with morphine on glia are mediated through a Beclin-1-dependent autophagy pathway (Rodriguez et al. 2017; Lapierre et al. 2018). Drugs with selective glial anti-inflammatory activity (i.e., ibudilast or AV411) attenuated the deleterious effects of HIV and opiate exposure, including HIV-1 replication, cytokine release, and neurotoxicity in vitro (El-Hage et al. 2014). Thus, the observed neuronal death is largely mediated by MOR-expressing glia (Zou et al. 2011), including astroglia (El-Hage et al. 2005, 2008b) and microglia (Turchan-Cholewo et al. 2008; Bokhari et al. 2009; Turchan-Cholewo et al. 2009; Gupta et al. 2010).

**Fig. 1:**
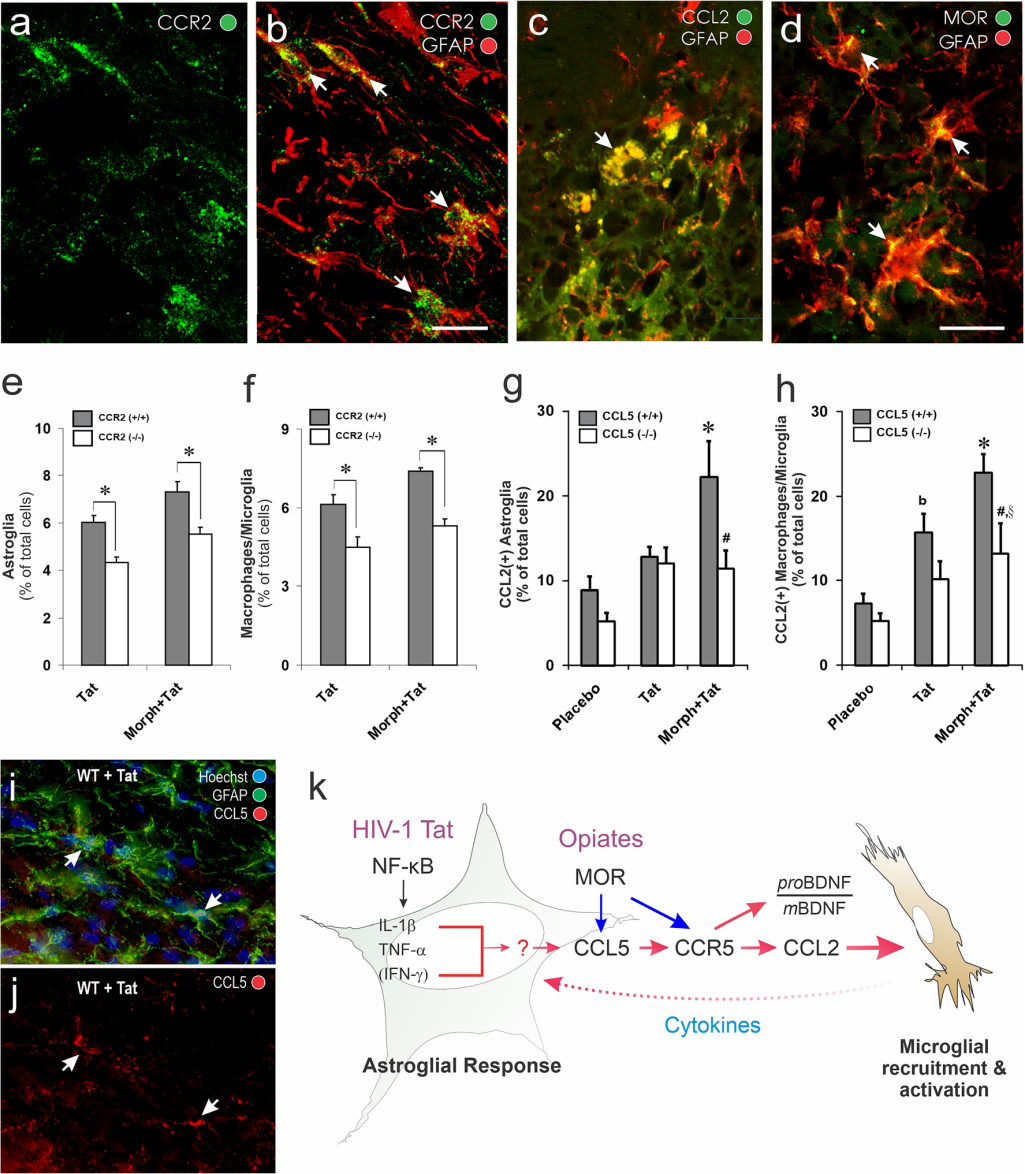
Opioids exacerbate HIV-1-induced CNS inflammation, in part, by augmenting CCL5-dependent increases in CCL2—key sites of opioid-HIV convergent interactions in glial inflammatory signaling cascades. Subpopulations of striatal glial fibrillary acidic protein (GFAP)-immunoreactive astrocytes in wildtype mice normally express CCR2 immunoreactivity (**a-b**; *arrows*), CCL2 (**c**; *arrow*), or μ-opioid receptor (MOR) (**d**; *arrows*) immunoreactivity (scale bars **a-b** = 25 μm; **c-d** = 15 μm). CCR2 deletion (−/−) significantly reduces HIV-1 Tat ± morphine-induced increases in GFAP+ astroglia (**e**) and F4/80+ macrophages/microglia (**f**) compared to wild type (+/+) mice at sites near (300 ± 100 μm) the site of Tat injection (*p < 0.05 vs. wild type mice) (see, El-Hage et al. 2006a). In wild-type mice, Tat ± morphine administration markedly increases the proportion of CCL2 immunoreactive astrocytes (**g**) or macrophages/microglia (**h**) [*p < 0.05 vs. other groups in wild-type or CCL5(−/−) mice; ^b^*p* < 0.05 vs. vehicle- or Tat plus morphine-treated wild-type mice; ^#^*p* < 0.05 vs. equivalent treatment in wild-type mice], while in CCL5 null mice, significant increases in CCL2 immunoreactivity were only seen in macrophages/microglia co-exposed to Tat and morphine (^§^*p* < 0.05 vs. vehicle injected CCL5 knockout mice) (see, El-Hage et al. 2008a). CCL5 expression in striatal GFAP-immunoreactive astrocytes (*arrows*) increases following Tat injections (**i, j**) compared to wild-type control mice (*not shown*) (El-Hage et al. 2008a). Opioids exacerbate HIV-1-induced CNS inflammation, in part, by increasing CCL5 and augmenting CCR5-dependent increases in CCL2 production by astrocytes resulting in the activation and recruitment of microglia/macrophages and spiraling inflammation (**k**). Additional factors likely mediate the proinflammatory cascade, but these are less well substantiated (*?*). Moreover, autocrine and reciprocal paracrine (*astroglial ↔ macrophage/microglial*) intercellular, feedback amplification mechanisms from macrophages/microglia are likely to be operative (*dashed red arrow*) (also see, Kang and Hebert 2011) and occur elsewhere within the cascade (*not shown*); effects of HIV-1 Tat/HIV, *red arrows*; sites of opioid convergence, *blue arrows*; pro-BDNF:mature BDNF (mBDNF) ratio (Kim et al. 2018). (**a-f**) Modified and reprinted with permission from El-Hage et al. (2006a). (**g-k**) Modified and reprinted with permission from El-Hage et al. (2008a)

The direct contributions of astrocytes to opioid and HIV interactions have been discussed previously (Dutta and Roy 2012; Hauser et al. 2012; Reddy et al. 2012; Hauser and Knapp 2014). Subsets of astroglia can express MOR, DOR and KOR (Stiene-Martin and Hauser 1991; Eriksson et al. 1992; Ruzicka et al. 1995; Gurwell et al. 1996; Hauser et al. 1996; Peterson et al. 1998; Stiene-Martin et al. 1998, 2001), as well as endogenous opioid peptides (Vilijn et al. 1988; Shinoda et al. 1989; Spruce et al. 1990; Hauser et al. 1990; Low et al. 1992). It appears that the ‘early’ events triggering the release of proinflammatory cytokines (i.e., TNF-α and IL-1β) from astroglia can be mediated by HIV Tat exposure alone (El-Hage et al. 2005, 2006a, b, 2008a). Opioids enhance HIV-1-induced inflammation later during the inflammatory cascade by exacerbating the sustained release of CCL5 from astrocytes, which subsequently triggers the release of CCL2 thereby enhancing the recruitment and activation of macrophages/microglia (El-Hage et al. 2008a) (Fig. 1). This is caused by the morphine-dependent exacerbation of Tat-induced increases in intracellular calcium concentration ([Ca^2+^]_i_) in astroglia (El-Hage et al. 2005)_,_ which accelerates the trafficking of NF-κB p65 (RelA) subunits to the nucleus and sustained CCL2, CCL5, and IL-6 transcription in astrocytes (El-Hage et al. 2008b).

#### Opioid and HIV Interactive Pathology in Microglia

Unlike in astrocytes, opiate and HIV interactions in microglia tend to be self-limiting (Turchan-Cholewo et al. 2009). Opiates initially trigger large increases in the production of proinflammatory cytokines (Hauser, unpublished), reactive oxygen (ROS) and nitrogen (RNS) species (Turchan-Cholewo et al. 2009), and the release of glutamate (Gupta et al. 2010) and ATP (Sorrell and Hauser 2014) extracellularly in Tat-exposed microglia. The release of glutamate is mediated by the catalytic subunit of the cystine-glutamate antiporter x_c_^−^ (xCT) (Gupta et al. 2010). Interestingly, following acute increases in the release of cytokines (e.g., TNF-α; unpublished), morphine no longer increases Tat-induced cytokine levels at 24 h; instead, their levels are reduced by opiate-dependent proteasome inhibition. The proteasome inhibitor, MG115, mimics the effects of morphine in decreasing proteasome activity at 24 h and blocks TNFα, IL-6, and CCL2 release from microglia, but does not increase ROS or RNS production (Turchan-Cholewo et al. 2009). The ubiquitin proteasome system (UPS) is typically viewed as contributing to opiate tolerance and physical dependence by modulating MOR downregulation (Massaly et al. 2014; Caputi et al. 2019), rather than MOR activity constraining the UPS. Thus, while HIV-exposed, MOR-expressing microglia show a burst of ROS and proinflammatory cytokine production in response to morphine, the cytokine release collapses within 24 h seemingly because sustained opiate exposure inhibits the UPS thereby preventing degradation of the IκB subunit and nuclear translocation of NF-κB (Turchan-Cholewo et al. 2009).

While neither astroglia nor microglia alone mimic the full inflammatory profile seen with opiates and HIV in the CNS; in combination, the neuroimmune signature more accurately mimics that seen in neuroHIV. Accordingly, we have proposed that opioids promote positive feedback through separate actions in astroglia and microglia in neuroHIV—resulting in spiraling inflammation and cytotoxicity (Hauser et al. 2005, 2007).

#### Opioid and HIV Interactive Pathology in Neurons

**Fig. 2:**
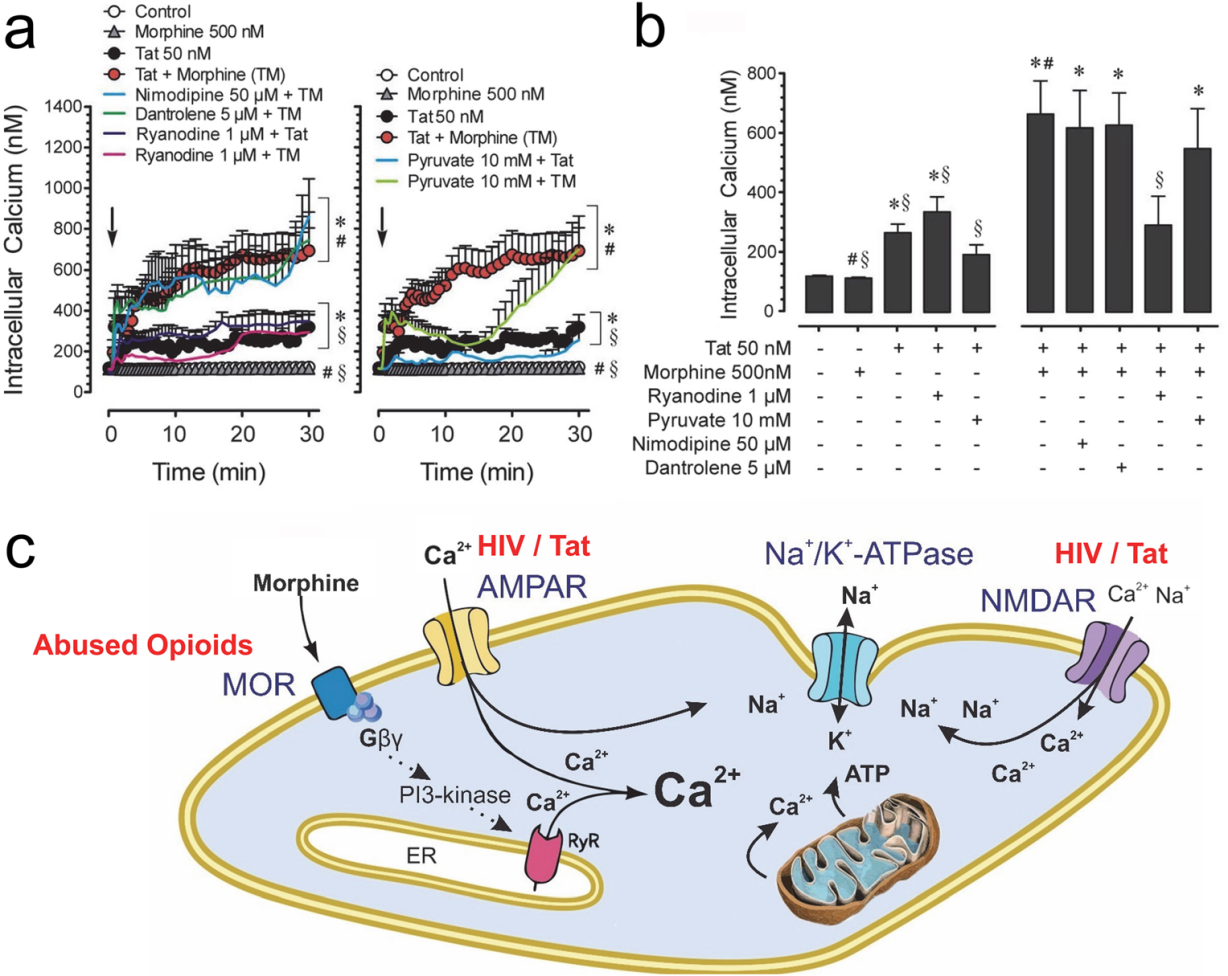
Morphine exacerbates the excitotoxic effects of HIV Tat by mobilizing Ca^2+^ from ryanodine (RyR)-sensitive internal stores. (**a**) Tat-induced increases in [Ca^2+^]_i_ were not attenuated by ryanodine, whereas ryanodine and pyruvate attenuate combined Tat and morphine-induced increases in [Ca^2+^]_i_. Nimodipine (L-type Ca^2+^ channel blocker) and dantrolene did not show any effects. (**b**) Average [Ca^2+^]_i_ over 10 min indicated ryanodine significantly blocked combined Tat and morphine-induced increases in [Ca^2+^]_i_, whereas no effects were noted for nimodipine, dantrolene, or pyruvate. *p < 0.05 vs. control, ^#^*p* < 0.05 vs. Tat 50 nM, ^§^*p* < 0.05 vs. TM, TM: Tat 50 nM + Morphine 500 nM. (**c**) Summary of HIV-1 Tat and morphine interactive neuronal injury in striatal medium spiny neurons. Combined Tat and morphine promotes structural and functional defects in dendrites via α-amino-3-hydroxy-5-methyl-4-isoxazolepropionic acid receptors (AMPAR), *N*-methyl-D-aspartic acid receptors (NMDAR), and MOR, causing influxes of Na^+^ and/or Ca^2+^, compensatory increases in Na^+^/K^+^-dependent ATPase activity, and a rapid loss in ATP mobilization with an inability to extrude excess Na^+^ via Na^+^/K^+^-ATPase caused by mitochondrial hyperpolarization. Dysregulation of [Ca^2+^]_i_ homeostasis by combined Tat and morphine appears to be mediated downstream of [Na^+^]_i_ at the level of calcium mobilization, which in turn appears to be regulated via ryanodine (RyR)-sensitive sites, and enhanced by morphine exposure likely via MOR-dependent stimulation of PI3-kinase and Ca^2+^ mobilization via the Gβγ protein subunit. (**a-b**) Modified and reprinted with permission from Fitting et al. (2014a)

Besides accentuating HIV-induced neurotoxicity via glial-mediated mechanisms, morphine appears to converge with HIV Tat to dysregulate ion homeostasis and dendritic injury through potential direct actions on neurons, even though some contributions of glia cannot be excluded in this study (Fitting et al. 2014a). Combined morphine and Tat exposure accelerates the formation of Tat-induced focal dendritic varicosities/swelling via a MOR-related mechanism that was caused by focal increases in Na^+^ influx and [Ca^2+^]_i_, an overload of Na^+^/K^+^-ATPase, ATP depletion, and a collapse in mitochondrial inner membrane potential (Fitting et al. 2014a). Importantly, morphine’s additive effects were mediated via a MOR-related mechanism, as the exacerbating effects of morphine were absent in neurons from MOR knockout mice, thus excluding TLR4 involvement (Fitting et al. 2014a). Further, morphine exacerbated Tat-dependent focal losses in ion homeostasis by mobilizing [Ca^2+^]_i_ through ryanodine-2 (RyR2)-sensitive sites (Fitting et al. 2014a) (Fig. 2). Although morphine typically acts via MOR in an inhibitory manner by activating G_i/o_-proteins (Sharma et al. 1977; Moises et al. 1994; Al-Hasani and Bruchas 2011), MOR-dependent stimulation of PI3-kinase and Ca^2+^ mobilization (Leopoldt et al. 1998) in neurons via the Gβγ protein subunit (Mathews et al. 2008) is presumed operative here (Fig. 2).

Glial-derived neuronal injury is not unidirectional. Neuronal damage-associated molecular patterns (DAMPs) and dysfunction can trigger both infected and uninfected glia to become reactive, resulting in further neuronal damage and escalating pathology. Neuronal injury can reactivate HIV in latently infected microglia (Alvarez-Carbonell et al. 2019). While the events underlying the disruption of neuronal-microglial activation that trigger the emergence of latent HIV are unclear, the induction of HIV expression appears to involve the production of DAMPs by injured neurons and can be turned “on”, e.g., by methamphetamine-induced sigma-1 (σ1) receptor activation, TNF-α and IL-1β, and TLR3 activation can be turned “off” by CX3CL1/fractalkine or glucocorticoid receptor activation (Alvarez-Carbonell et al. 2017, 2019).

### Neural Systems Selectively Disrupted by Opiate and HIV Interactions

#### Blood-Brain Barrier and the Neurovascular Unit

Despite growing evidence on how opiates and HIV interact to impact the neuropathology of HIV, little is known about their interactive effects on the blood-brain barrier (BBB). BBB integrity and function are critical for maintaining CNS homeostasis, and mediating neuroimmune interactions with the periphery and drug delivery into the CNS. HIV and many individual HIV proteins can breakdown the BBB disrupting tight junction proteins (Dallasta et al. 1999; Boven et al. 2000; Andras et al. 2003; Mahajan et al. 2008; Banerjee et al. 2010; Gandhi et al. 2010; Xu et al. 2012; Patel et al. 2017) and decreasing transendothelial electrical resistance (TEER) (an in vitro measure of barrier integrity) (Mahajan et al. 2008; Gandhi et al. 2010; Mishra and Singh 2014; Patel et al. 2017), with resultant paracellular “leakage” of compounds/current between compromised barrier endothelial cells (Mahajan et al. 2008; Gandhi et al. 2010; Wen et al. 2011; McLane et al. 2014; Leibrand et al. 2017, 2019). Although opioids can also impair the BBB through alterations in tight junction proteins and/or increased paracellular flux (Baba et al. 1988; Mahajan et al. 2008; Wen et al. 2011; Leibrand et al. 2019), others have found that it is morphine withdrawal, not the continued exposure to morphine, that most greatly disrupts BBB integrity (Sharma and Ali 2006). In addition to perturbing paracellular dynamics, morphine may also alter the expression and/or function of drug efflux proteins, such as P-glycoprotein (P-gp). Sub-chronic and chronic morphine exposure is reported to increase P-gp expression and/or function (Aquilante et al. 2000; Mahajan et al. 2008; Yousif et al. 2008; Leibrand et al. 2019). Alternatively, other investigators report no changes in P-gp with chronic exposure (Chaves et al. 2016), while some see increases upon morphine withdrawal (Yousif et al. 2012; Chaves et al. 2016). Alterations in drug transport proteins would impact the central accumulation and efficacy of therapeutic drugs that are their substrates.

**Fig. 3:**
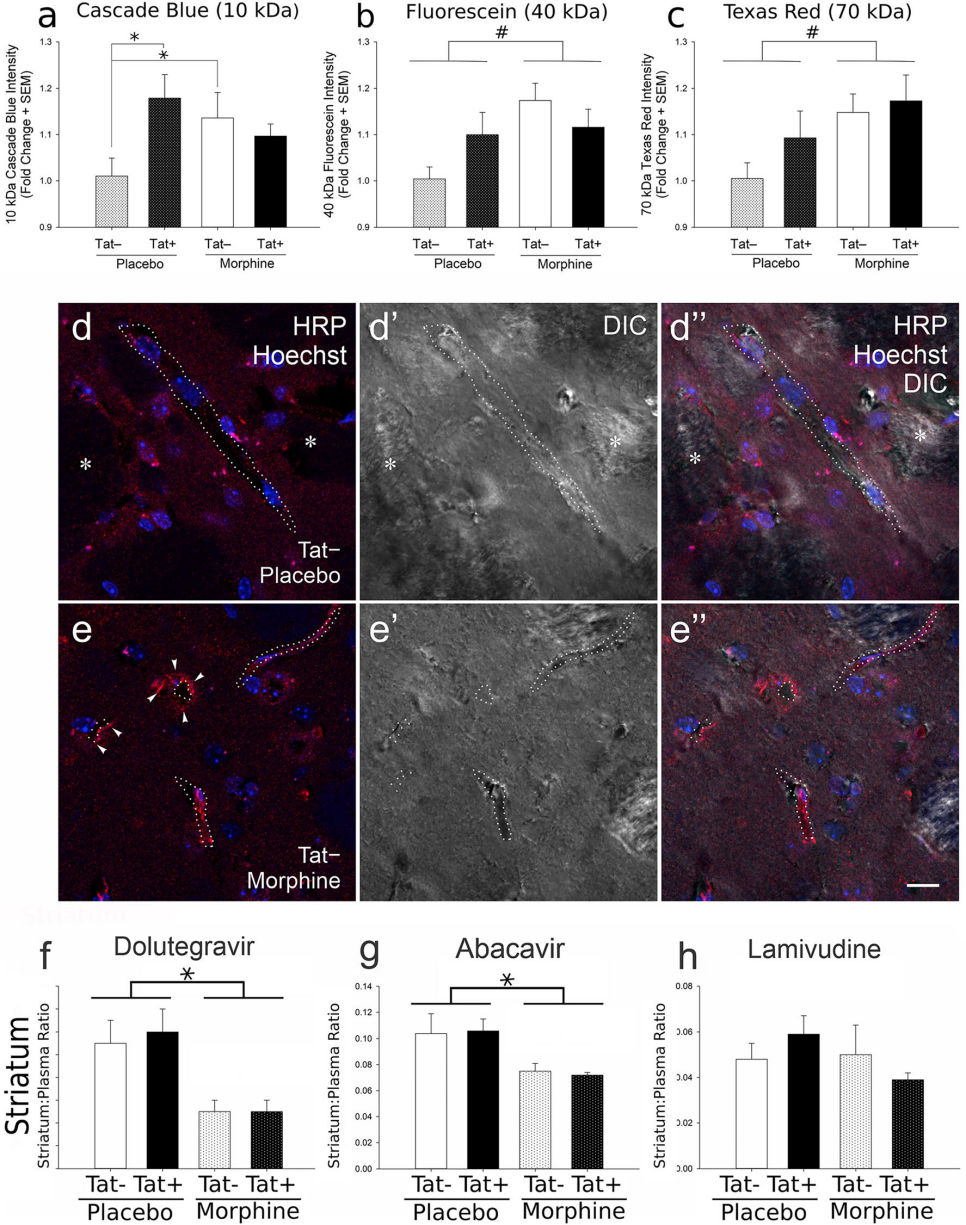
Effects of HIV-1 Tat and morphine on BBB leakiness and on antiretroviral brain concentrations. After 14 days of Tat induction, there was a significant increase in the 10 kDa (Cascade Blue®) tracer leakage into the brain in Tat + placebo as compared to Tat − placebo mice (*p < 0.05) and in Tat − mouse brains upon exposure to morphine as compared to Tat − placebo mice (*p < 0.05) (**a**). There was a significant main effect of morphine, resulting in reduced integrity of the BBB and increased leakage of the higher molecular weight (40 kDa and 70 kDa) tracers in morphine-exposed groups as compared to the those groups (Tat + and Tat − together) not exposed to morphine (placebo) (^#^*p* < 0.05; significant main effect of morphine) (**b, c**). Data represent the fold change in mean fluorescence intensity ± SEM; *n* = 8 Tat−/placebo, *n* = 6 Tat+/placebo, *n* = 9 Tat−/morphine, and *n* = 7 Tat+/morphine mice. Additionally, morphine exposure increased horseradish peroxidase (HRP) extravasation from the vasculature into the perivascular space and/or parenchyma in the striatum (**d, e**). HRP antigenicity was detected by indirect immunofluorescence (red) in tissue sections counterstained with Hoechst 33342 (blue) to reveal cell nuclei and visualized by differential interference contrast (DIC)-enhanced confocal microscopy. HRP extravasation into the striatal perivascular space/parenchyma was especially prevalent in morphine-exposed mice (arrowheads; left-hand panels in **e** versus **d**). The dotted lines (············) indicate the approximate edge of the capillaries/post-capillary venules; while intermittent dotted lines (· · · · · · ·) indicate the approximate edge of a partly sectioned blood vessel that appears partially outside the plane of section. The asterisks (*) indicate white matter tracts within the striatum. Representative samples from ≥ *n* = 4 mice per group. All images are the same magnification. Scale bar = 10 μm. Antiretroviral tissue-to-plasma ratios in striatum (**f–g**). Irrespective of Tat exposure, morphine significantly reduced the levels of dolutegravir (**f**) and abacavir (**g**), but not lamivudine (**h**), within the striatum, as compared to placebo. (* *p* < 0.05; main effect for morphine). Data represent the tissue-to-plasma ratios ± SEM sampled from *n* = 9 Tat−/placebo, *n* = 9 Tat+/placebo, *n* = 6 Tat−/morphine, and *n* = 8 Tat+/morphine mice. (**a–h**) Modified and reprinted with permission from Leibrand et al. (2019)

Using a primary human brain microvascular endothelial cell (BMEC) and astrocyte co-culture model, Mahajan et al. (2008) were among the first to demonstrate that co-exposure to morphine and HIV-1 Tat resulted in greater increases in TNF-α and IL-8 levels and decreases in barrier tightness (measured by TEER) than either morphine or Tat alone. Morphine and Tat co-exposure also additively increased JAM-2, while zonula occludens-1 (ZO-1) levels were decreased by morphine or by Tat individually, and occludin protein levels were decreased by morphine alone but not Tat (Mahajan et al. 2008). Using the inducible Tat transgenic mouse model, Leibrand et al. (2019), also demonstrated that HIV-1 Tat and morphine act independently to disrupt BBB integrity. In these studies, morphine, and to a lesser extent Tat, exposure increased the leakage of fluorescently labeled dextrans from the circulation into the brain (Leibrand et al. 2017, 2019) (Fig. 3). Morphine exposure decreased the penetration of select ARVs in the brain, in a region-specific manner (Leibrand et al. 2019) (Fig. 3). Morphine exposure also resulted in increased expression and function of the drug efflux transport protein, P-gp, suggesting a mechanism by which morphine decreased the ARV concentrations (Leibrand et al. 2019). This finding suggests that morphine exposure could impact the efficient delivery of any therapeutic drug that is a substrate of P-gp into the CNS. Future research should also investigate morphine’s impact on other drug transport proteins important for ARV delivery to the brain.

HIV, HIV-1 viral proteins, and opiate-induced barrier dysfunction is associated with increased infiltration of monocyte-derived macrophages (MDMs) into the brain. Enhanced influx of peripheral (infected) macrophages into the brain can serve to replenish viral reservoirs and further promote neuroinflammation. Several studies have examined the individual impact of HIV, Tat, or morphine on monocyte adhesion or migration into the CNS (Nottet et al. 1996; Wu et al. 2000; Fischer-Smith et al. 2001; Pello et al. 2006; Williams et al. 2013a, 2014; Strazza et al. 2016; Leibrand et al. 2017; Chilunda et al. 2019). However, fewer studies have examined the combined effects of HIV/Tat and opiates. Co-exposure of HIV-1 Tat and morphine on astrocytes increases the production of chemoattractants, primarily CCL2 and CCL5, and increases microglial migration. These effects were inhibited by MOR blockade (El-Hage et al. 2006b). Co-exposure of Tat and morphine or buprenorphine to a BBB model increases monocyte transmigration in response to CCL5 and other chemokines (Mahajan et al. 2008; Jaureguiberry-Bravo et. al. 2016). In *S. pneumoniae*-infected mice, morphine and/or Tat exposure significantly enhances immune cell trafficking into the brain via actions at TLR2 and TLR4 (Dutta and Roy 2015).

**Fig. 4:**
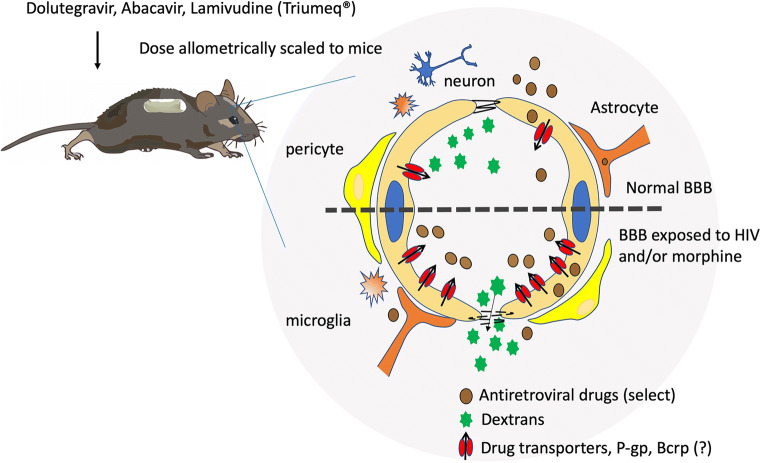
Schematic representation of the blood-brain barrier and other components of the neurovascular unit. Under normal conditions (represented above the dotted line), tight junctions are intact which restricts the leakage of paracellular, typically small hydrophilic, compounds, across the barrier and into the brain. Additionally, there is a basal expression of efflux transporters, such as P-glycoprotein (P-gp), which effluxes substrates out of the brain, serving to restrict overall accumulation within the brain. In the setting of HIV and opiate exposure (represented below the dotted line), there is a breakdown of the tight junction proteins and increased leakage of paracellular compounds into the brain. Additionally, opiate exposure increases efflux transporter expression, including P-gp and potentially breast cancer resistance protein (Bcrp), thereby restricting overall brain penetration of drugs (like many antiretroviral drugs) which are substrates for these transporters and in response to HIV and/or opioid exposure.

Taken together, BBB damage from HIV and/or opiates can disrupt the homeostasis within the brain. Breakdown of paracellular processes, through decreases in tight junction proteins and increased cellular adhesion proteins, increased leakage of circulating molecules into the brain and increased monocyte/MDM adhesion and transmigration into the brain, which if infected, can serve to replenish viral reservoirs within the CNS. Furthermore, alterations in drug transport proteins within the brain can decrease ARV efficacy by decreasing drug concentrations. Collectively, these changes serve to maintain HIV infection within the brain (see Fig. 4**;** Tables 1 and 2).

#### White Matter/Oligodendroglial Pathology

HIV can cause white matter damage (Gosztonyi et al. 1994; Langford et al. 2002; Xuan et al. 2013) even with less severe forms of HAND (Chen et al. 2009; Leite et al. 2013; Correa et al. 2015). Diffusion tensor magnetic resonance imaging (DTI) demonstrates white matter damage early in HAND (Ragin et al. 2004; Stubbe-Drger et al. 2012; Leite et al. 2013; Correa et al. 2015). White matter deficits are associated with cognitive impairment, including shortfalls in memory (Ragin et al. 2005), executive function (Correa et al. 2015), motor speed (Wu et al. 2006; Stubbe-Drger et al. 2012), and perhaps depression (Schmaal and van Velzen 2019). Preclinical studies in simian immunodeficiency virus- (SIV-) infected rhesus macaques (Marcario et al. 2008) and HIV-infected humanized mice (Boska et al. 2014) support the clinical findings. Injury to oligodendrocytes (OLs) can occur very early in the disease (see review, Liu et al. 2016b). Viral proteins, including Tat, gp120, and Nef, have been implicated in OL injury in vitro (Kimura-Kuroda et al. 1994; Bernardo et al. 1997; Radja et al. 2003; Nukuzuma et al. 2012; Zou et al. 2015), and in animal models in vivo (Radja et al. 2003; Hauser et al. 2009; Zou et al. 2015). Importantly, Tat has been detected in OLs in the brains of AIDS patients (Del Valle et al. 2000).

HIV likely damages OLs through both direct and indirect actions. OLs lack CD4, and reports of OL infection by HIV are variable (Esiri et al. 1991; Albright et al. 1996; Wohlschlaeger et al. 2009); thus, HIV infection of OLs is unlikely a major avenue of OL or white matter damage (discussed below). Alternatively, bystander damage to OLs through the production of “virotoxins” and “cellular toxins” (Nath 1999) by infected neighboring cells is more likely to be operative (Hauser et al. 2009; Zou et al. 2015; Jensen et al. 2019; Zou et al. 2019). ARVs also contribute to OL cytotoxicity (Jensen et al. 2015; Festa et al. 2019; Jensen et al. 2019). HIV-1 Tat directly induces damage in isolated OLs through α-amino-3-hydroxy-5-methyl-4-isoxazolepropionic acid (AMPA)/*N*-methyl-D-aspartic acid (NMDA) receptor-dependent mechanisms (Zou et al. 2015) and is also associated with abnormal Kv1.3 activity (Liu et al. 2017). Immature OLs are preferentially targeted by Tat compared to differentiated OLs (Khurdayan et al. 2004; Hahn et al. 2012; Zou et al. 2015, 2019). While the reasons why immature OLs are more susceptible to Tat are unclear, unlike mature OLs, Tat preferentially upregulates GSK-3β signaling in undifferentiated OLs by inhibiting Ca^2+^/calmodulin-dependent protein kinase II β (CaMKIIβ) (Zou et al. 2019).

Opioid abuse by itself can result in demyelination, leukoencephalopathy, and lesions in white matter (Offiah and Hall 2008; Eran and Barak 2009; Morales Odia et al. 2010; Bora et al. 2012; Li et al. 2013), and the degree of myelin disruption correlates with the duration of opiate dependence (Ivers et al. 2018). Chronic oxycodone exposure in rats causes some axonopathies and reduces the size of axonal fascicles, decreases myelin basic protein levels, and causes the accumulation of amyloid-β precursor protein (APP) (Fan et al. 2018). Most preclinical studies have examined the effects of opioids and opioid receptor blockade on OL maturation and/or the timing of myelination (Hauser et al. 1993; Knapp et al. 1998; Stiene-Martin et al. 2001; Sanchez et al. 2008; Knapp et al. 2009; Vestal-Laborde et al. 2014). OLs can transiently express MORs and other opioid receptor types (Knapp et al. 1998; Tryoen-Toth et al. 2000; Knapp et al. 2001; Stiene-Martin et al. 2001). Selective MOR and possibly KOR activation can directly modulate the growth of OLs in vitro (Knapp and Hauser 1996; Knapp et al. 1998, 2001).

Despite long-standing evidence of white matter damage early during the infection even in asymptomatic PWH (Price et al. 1988; Gray et al. 1996; Chen et al. 2009; Stubbe-Drger et al. 2012; Jensen et al. 2019), few studies have examined how opiate exposure affects OLs and myelin in neuroHIV (Tables 1 and 2). Increased demyelination is reported in SIV-infected rhesus macaques chronically treated with morphine (4× daily, up to 59 weeks) (Marcario et al. 2008). Specifically, morphine-treated SIV macaques developed more subtle, focal, dysmyelinating lesions, with accumulations of macrophages in areas of myelin loss (Marcario et al. 2008), as well as accompanying gliosis (Marcario et al. 2008; Rivera-Amill et al. 2010a; Bokhari et al. 2011). Morphine exposure increased degeneration of OLs in Tat+ mice, which was accompanied by elevations in caspase-3 activation and TUNEL reactivity in OLs and reversible by naloxone or naltrexone, respectively (Hauser et al. 2009). Although OLs can express MOR both in vivo (Stiene-Martin et al. 2001) and in vitro (Hauser et al. 2009), it remains unclear the extent to which MOR activation in OLs directly mediates HIV pathogenesis.

#### Neural Progenitors as an HIV Reservoir and Target for Opioids

Even though neural progenitors (Krathwohl and Kaiser 2004; Lawrence et al. 2004; Rothenaigner et al. 2007; Schwartz et al. 2007; Balinang et al. 2017), neuroblast cell lines (Ensoli et al. 1994; Rothenaigner et al. 2007), and/or immature astroglia (Atwood et al. 1993; Tornatore et al. 1994; Barat et al. 2018) can harbor HIV infection (reviewed by Hauser and Knapp 2014; Putatunda et al. 2019), the degree to which they are a source of active infection or serve as a latent viral reservoir (Blankson et al. 2002; Bruner et al. 2019) by retaining intact proviral DNA within incipient macroglial progeny is uncertain. In fact, spurious reports of HIV-infected adult neurons (Torres-Munoz et al. 2001; Canto-Nogues et al. 2005) may result from the retention of proviral genes that integrated into pluripotent neural progenitors or neuroblasts at earlier stages during maturation. Importantly, prolonged exposure to opioids can increase the production of HIV in human neural progenitor cells (hNPCs). Exposure of R5-tropic HIV_BaL_-infected hNPCs to morphine continuously for 21 d increased viral production compared to HIV_BaL_ infection alone in vitro (Balinang et al. 2017).

Besides being able to infect hNPCs, HIV may also affect their maturation and the fate of neural stem cells. That HIV or gp120 can inhibit adult neurogenesis (Okamoto et al. 2007; Lee et al. 2013; Putatunda et al. 2018) has been the topic of past reviews (Schwartz and Major 2006; Venkatesan et al. 2007; Peng et al. 2008, 2011; Ferrell and Giunta 2014; Hauser and Knapp 2014; Putatunda et al. 2019). How HIV inhibits the self-renewal, tripotential differentiation, and survival of neural progenitors/stem cells or the genesis of adult neurons in the subgranular zone (SGZ) of the dentate gyrus is uncertain. HIV and gp120 [via actions at the same chemokine receptor(s) (Tran and Miller 2005; Li and Ransohoff 2008)] are proposed to modulate the adult neurogenesis via Notch (Fan et al. 2016), by obstructing a cell cycle checkpoint via the activation MAPK-activated protein kinase 2 and Cdc25B/C (Okamoto et al. 2007), or through signaling by platelet-derived growth factor BB (Chao et al. 2014) or BDNF (Lee et al. 2013). The extent that HIV regulates the genesis of neural progenitors within the subventricular zone of the developing CNS through similar mechanisms as in the adult SGZ of the dentate gyrus is uncertain—even though HIV disrupts the generation of neurons and glia during maturation or in adults. For example, MAPK/ERK1/2 enhances p53- and p21-dependent downregulation of cyclin D1 hindering progression through the G_1_ phase of the cell cycle in hNPCs (Mishra et al. 2010; Malik et al. 2014). Importantly, opioids too can affect the genesis of neurons and glia during maturation or in the adult directly via convergent pathways (Hauser and Knapp 2018; Kibaly et al. 2018) suggesting yet another site of opioid and HIV interactions in dysregulating the creation and fate of new neurons and glia.

Few studies have examined the interplay between opioids, neural progenitors and HIV/HIV proteins. Sustained exposure (4 d) to morphine (500 nM) and Tat_1–72_ (100 nM) decreased the viability of MOR-expressing striatal glial precursors, and to a lesser extent astrocytes, and this coincided with caspase-3 activation (Khurdayan et al. 2004). By contrast, comparably administered morphine or Tat alone was sufficient to decrease the viability of immature glia/glial progenitors in spinal cord cultures, while Tat and morphine failed to interact (Buch et al. 2007). Collectively, these findings were the first to indicate that opioid and/or Tat could enhance programmed cell death in subpopulations of glial precursors in a developmentally regulated and region-dependent manner (Khurdayan et al. 2004; Buch et al. 2007). In human glial progenitors, co-administering morphine (500 nM) increased the antiproliferative effects of Tat (12–48 h) or conditioned medium from HIV-1_SF162_-infected MDMs (12 h), while paradoxically reversing the antiproliferative effects from HIV-1_IIIB_ conditioned medium (12 h) (Hahn et al. 2012). In these studies, Tat or HIV exposure reduced the proliferation of Sox2+ and Olig2+ undifferentiated glial and oligodendroglial progenitors, respectively, while progenitor viability was unchanged (Hahn et al. 2012). In human neural progenitor cells (hNPCs), sustained infection with R5-tropic HIV_BaL_ increased the proliferation and premature differentiation of hNPCs into both neurons and astrocytes, and both measures were significantly enhanced by morphine co-exposure (Balinang et al. 2017). Importantly, immunoneutralizing antibodies (Hahn et al. 2012) or the selective antagonist, maraviroc (Balinang et al. 2017), were able to significantly attenuate the consequences of R5-tropic HIV infection on hNPC differentiation and fate confirming a direct role of CCR5 in these processes. Lastly, decreases in the proliferation of hNPCs seen with morphine and Tat are, in part, regulated by ERK1/2-dependent increases in p53 and p21 expression (Malik et al. 2014) and can be modulated by PDGF BB suggesting a possible therapeutic target (Malik et al. 2011). Thus, morphine can exaggerate R5-tropic HIV-induced alterations in the maturation and fate of human and rodent NPCs, thereby further disrupting the balance of neural cell types and CNS function.

## Matters Needing Further Consideration in Opioid and HIV Comorbidity

The interplay of complex host and viral genetic differences is likely to play a huge role in determining pathologic outcomes in PWH. For example, differences in HIV strains/variants (Rao et al. 2013) and human/host genetic variability (Proudnikov et al. 2012), pharmacokinetics (Kuhlman et al. 1996; Eap et al. 2002; Elkader and Sproule 2005; Kharasch 2017; Kringen et al. 2017), and sex (Zubieta et al. 2002; Taylor and Davies 2010; Venuto et al. 2014; Marinho et al. 2019) all contribute to variability in responsiveness. The following subsections will focus on key factors affecting opioid and HIV comorbidity.

### HIV-1 Genetics

Genetic differences among HIV-1 variants have a significant impact on HIV transmission, disease progression, as well as the response to ARV therapy (see reviews, Geretti 2006; Taylor et al. 2008; Tyor et al. 2013; Tables 1 and 2). Pre-cART studies provide substantial evidence that HIV clade differences can influence HAND (Gupta et al. 2007; Sacktor et al. 2009; Boivin et al. 2010; McArthur et al. 2010; Rao et al. 2013), with HAND severity being highest for clade D and B strains, followed by C and A clades (Tyor et al. 2013). These findings are supported by preclinical studies in which clade B or clade C HIV-infected macrophages were intracranially injected into severe combined immunodeficient mice (SCID) mice. Exposure to clade B isolates induced more severe memory deficits, as well as greater astrogliosis and neuronal damage (Rao et al. 2008, 2013). In another example, the Tat dicysteine motif (CC) at positions 30 and 31, which is commonly found in clade B isolates, appears to worsen HAND (Mishra et al. 2008; Rao et al. 2013) and has been studied extensively in vitro (Ranga et al. 2004; Rao et al. 2008; Zou et al. 2011; Krishnan and Chatterjee 2015). Clade B Tat is more intrinsically cytotoxic to primary neurons in vitro than clade C Tat (Li et al. 2008; Campbell et al. 2011; Zou et al. 2011), resulting in increased proinflammatory cytokine production (e.g., IL-6 and TNF-α) (Gandhi et al. 2009) and monocyte recruitment/migration into the brain (Ranga et al. 2004; Rao et al. 2008), and increased disruption of the BBB (Gandhi et al. 2010). Similarly, the production of the inflammatory mediators prostaglandin E_2_ and the thromboxane A_2_ receptor by astrocytes is more significantly increased by clade B than clade C gp120 (Samikkannu et al. 2011). Sequence and structural alterations in gp120 have been demonstrated between clades B and C (Gnanakaran et al. 2007) and potentially contribute to these observed differences.

When considering effects of HIV clade variants in the presence of opioids, the overall toxicity in MSNs seen with clade C Tat (30% neuronal losses) was considerably less than with clade B (70% losses) (Zou et al. 2011). Although clade B HIV predominates in Western countries, future clinical longitudinal studies are necessary that employ HIV clade testing in HIV-1 infected opioid users to confirm the hypothesis that opioid interactive effects on HAND pathogenesis depend on the HIV clade assessed.

Besides HIV genetic diversity, differences in HIV tropism add another level of complexity. Morphine interactions can differ significantly between X4 and R5-tropic gp120 variants depending on the outcome measure (El-Hage et al. 2011b; Podhaizer et al. 2012; Balinang et al. 2017; Kim et al. 2018). Increased infectivity in the presence of morphine was noted for the R5-tropic HIV-1_SF162_ strain in a human hepatoma Huh7.5.1 cell line model, whereas the infectivity rate with the X4-tropic HIV-1_LAI/IIIB_ strain was unaffected by morphine (El-Hage et al. 2011b).

To date, no clinical studies have assessed whether opioid interactions with R5- or R4-preferring HIV strains differentially impact the severity of HAND. However, the findings from preclinical studies indicate that HIV-1 strain-specific differences are critical determinants in shaping both the timing and pattern of neurotoxic interactions with opioid drugs.

### Host Genetics

Host genetic variability can be a major determinant in individual susceptibility to HIV infectivity and may influence neuroHIV progression in the context of opiate co-exposure. The importance of CCR5 for HIV infectivity and polymorphisms in this gene are well established. Individuals who are homozygous in the CCR5 gene (CCR5Δ32) are highly resistant to infection by CCR5- (R5-) tropic HIV as demonstrated by individuals heterozygous for CCR5Δ32 who display partial resistance to infection and slower disease progression (Huang et al. 1996; Liu et al. 1996; van Rij et al. 1999). Besides CCR5, polymorphisms of other chemokine co-receptors and/or their cognate ligands have been implicated in HIV infectivity, including CCR2 (Smith et al. 1997; Kostrikis et al. 1998), CCL5 (Liu et al. 1999; McDermott et al. 2000), and CXCL12 (Winkler et al. 1998). Authoritative reviews on other gene polymorphisms that modify HIV infectivity and disease progression have been published (Lama and Planelles 2007; Singh and Spector 2009; Chatterjee 2010; Aouizerat et al. 2011).

Gene polymorphisms of opioid (*OPRM1* and *OPRK1*) and non-opioid (e.g., *DRD1* and *DRD2*) drug/neurotransmitter receptor genes are associated with altered HIV infectivity, viral loads and CD4+ cell counts (Proudnikov et al. 2012; Regan et al. 2012; Jacobs et al. 2013; Proudnikov et al. 2013; Dever et al. 2014). Not only do MORs mediate the behavioral consequences of opiate abuse (Bond et al. 1998; Szeto et al. 2001; Ikeda et al. 2005; Kreek et al. 2005; Xu et al. 2014b), but the ability of MOR to modulate HIV chemokine co-receptor signaling through cross desensitization or through direct molecular interactions suggest MOR may influence HIV infectivity at multiple levels. The unique ability of MOR to modulate HIV co-receptor function, prompted inquiry regarding whether variants of the *OPRM1* gene (polymorphisms or splicing variants) might differentially effect HIV infectivity and/or opiate addictive behaviors. In a sample of 1031 HIV-1-infected women, 18 *OPRM1* polymorphisms were significantly associated with decreases or increases in HIV infectivity and responsiveness to cART (Proudnikov et al. 2012). Other gene polymorphisms, such as enzymes affecting drug metabolism (Meyer and Zanger 1997; Benowitz et al. 2006) and other neurochemical systems (Herman and Balogh 2012; Koob and Volkow 2016) can also affect drug dependence. The A118G variant of *OPRM1* alters the regulation of proinflammatory cytokine secretion (i.e., TNF-α, IL-10, IFN-γ) from peripheral immune cells (Matsunaga et al. 2009). Overall, these findings suggest that polymorphisms in MOR ligands/genes (*OPRM1*) can influence the pathophysiology of HIV-1.

Nineteen different *OPRM1* spliced variants have been described in humans (Pasternak 2004, 2014; Xu et al. 2014a; Lu et al. 2015). *OPRM1* alternative splicing may also influence susceptibility to HIV-1 infection (Dever et al. 2012, 2014). Although many variants are thought to be non-functional and fail to traffic from the endoplasmic reticulum, increasing evidence suggests they may oligomerize other G Protein-coupled receptors or bind chaperones to assist in trafficking to the plasma membrane (Samoshkin et al. 2015; Zhu et al. 2019). Quantitative and qualitative differences in human MOR splice variant expression levels have been noted across different CNS cell types following exposure to HIV (Dever et al. 2012, 2014). Interestingly, an excitatory, MOR-1 K splice variant, that couples to Gα_S_ (Gris et al. 2010) is preferentially expressed in human astroglia (Dever et al. 2012) and has been shown to correlate with HIVE and cognitive impairment (Dever et al. 2012, 2014).

### MOR and Chemokine Receptor Interactions (CCR5, CXCR4)

The ability of opiates to modulate HIV infection and HIV neuropathogenesis/disease progression may be partly due to the interactive effects seen between the opioid and chemokine receptors, specifically MOR and CCR5 or CXCR4 (Rogers and Peterson 2003; Steele et al. 2003; Szabo et al. 2003; Festa and Meucci 2012). The potential mechanisms for this interaction can include heterologous cross-desensitization via downstream signaling (Rogers et al. 2000; Steele et al. 2002; Song et al. 2011) and/or potentially via direct opioid-chemokine receptor dimeric or heteromeric interactions (Suzuki et al. 2002; Chen et al. 2004; Nash and Meucci 2014). MOR and DOR activation can heterologously desensitize CCR5 responsiveness to CCL3, CCL4, and CCL5 in monocytes (Grimm et al. 1998; Szabo et al. 2003; Chen et al. 2004). The cross-desensitization appears to be regulated by MOR-dependent PKCζ activation and CCR5 phosphorylation and downregulation (Song et al. 2011). Alternatively, MOR-induced downregulation of CCL2 and CCL4 mRNA reciprocally upregulates the expression of their associated receptors, CCR2b, CCR3, and CCR5 (Mahajan et al. 2005). A previous study reported significant upregulation of CCR5 and CXCR4 expression in CD14 monocytes with [D-Ala^2^, N-MePhe^4^, Gly-ol]-enkephalin (DAMGO), a MOR ligand, exposure with enhanced replication of both X4- and R5-tropic viral strains of HIV (Steele et al. 2003). For CXCR4, bidirectional heterologous desensitization is less evident with MOR but has been reported for KOR, with Ca^2+^ signaling experiments suggesting that cross-desensitization occurs within seconds of KOR or CXCR4 activation in a concentration-dependent manner (Finley et al. 2008). Thus, opiates acting at different opioid receptors in the presence of HIV appear to activate chemokine receptor signaling and can contribute to the synergistic effects of HIV and opioid drug co-exposure seen in neuroHIV progression.

The ability of opiates to modulate CCR5 expression in the CNS has been demonstrated to occur in various cell types, including microglia (Bokhari et al. 2009), and astrocytes (Mahajan et al. 2002). Specifically, in astrocytes MOR activation enhanced CCR5 and additional HIV-1 entry co-receptor (CCR3 and CXCR2) expression, whereas local production of HIV-1 protective chemokines (IL-8, CCL4) was inhibited (Mahajan et al. 2002). Deletion of CCR5 significantly attenuates morphine-induced increases in astrocyte CCL2 immunoreactivity in Tat transgenic mice (El-Hage et al. 2008a) (Fig. 1). Interestingly, the proportion of CCL2 immunoreactive macrophages/microglia in CCL5(−/−) mice after Tat and morphine co-administration still showed a significant upregulation, suggesting CCL5 regulates Tat and morphine-induced increases in CCL2 in astrocytes, but not in microglia (El-Hage et al. 2008a) (Fig. 1).

**Fig. 5:**
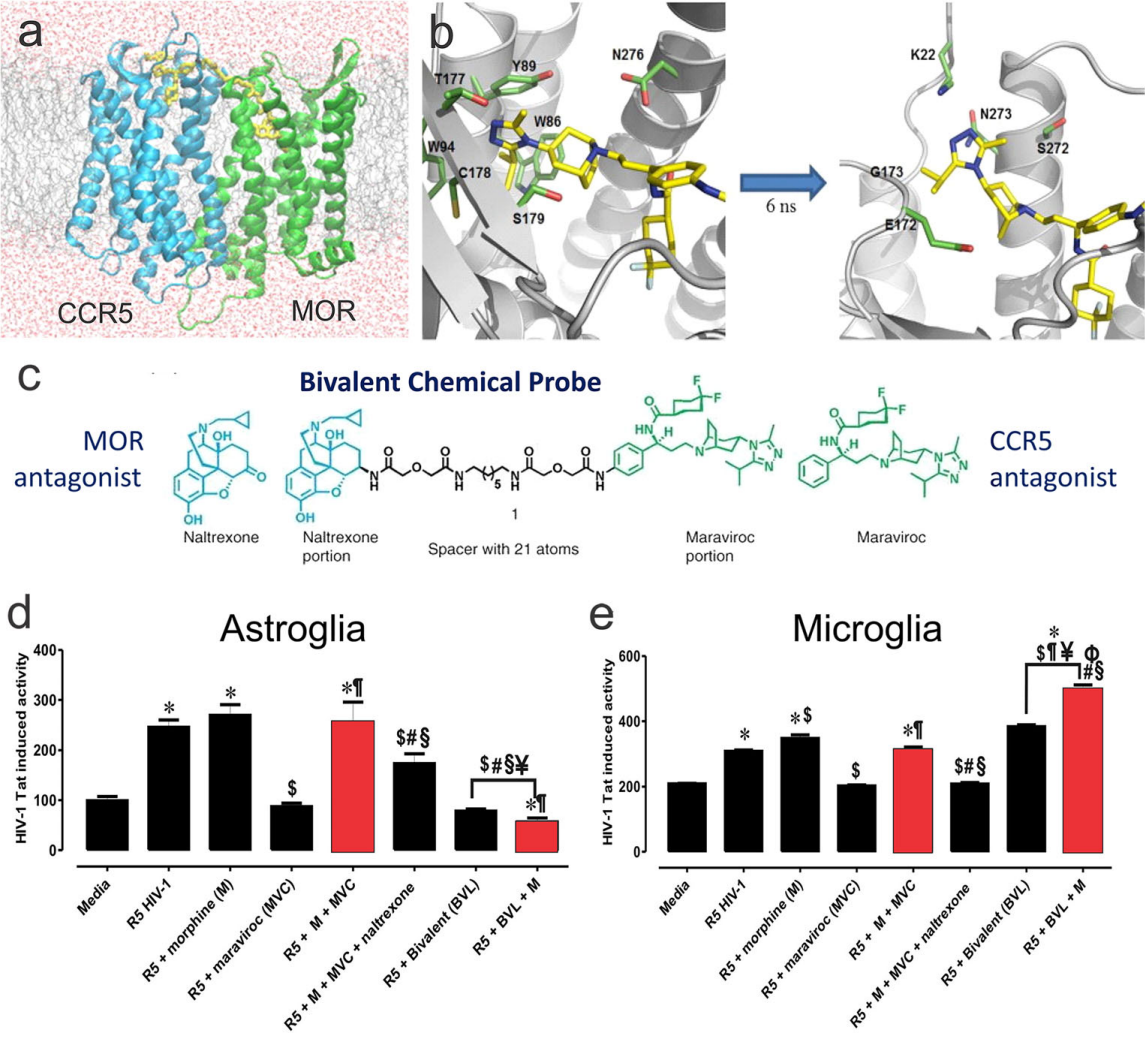
Differential inhibition of HIV-1 entry into human glia by maraviroc and a bivalent CCR5-MOR antagonist (BVL) with cell-specific interactions in combination with morphine. (**a**) Construction of a MOR-CCR5 heterodimer model in a membrane (*gray*), and aqueous surrounds (*red*) system. The green protein represents MOR and the blue protein represents CCR5, while the bivalent ligand is colored in yellow. (**b**) Different binding pocket (green) for the triazole moiety of the bivalent ligand yellow) at 0 ns and 6.0 ns. (**c**) Construction of a chemical probe that interacts with both the MOR and CCR5 receptors simultaneously. To monitor HIV-1 infection (**d**) astrocytes and (**e**) microglia were transfected with a pBlue3′LTR-luc reporter sensitive to Tat expression and luciferase activity was measured. Data indicate that maraviroc’s antiviral effects are completely negated in both astrocytes and microglia when morphine is present (*red bars*). Interestingly, unlike maraviroc, the bivalent compound blocked HIV entry in astrocytes irrespective of morphine treatment. By contrast, the bivalent antagonist exacerbated HIV infectivity in microglia in the presence of morphine (*red bars*). The findings reveal fundamental differences in co-regulation of MOR and CCR5 expression in astroglia and microglia upon HIV and/or morphine exposure (see El-Hage et al. 2013). Values are luminescence intensity ± SEM from 3 to 5 independent experiments at 18 h post-infection (*p < 0.005 vs. un-infected cells; ^$^*p* < 0.05 vs. R5 HIV-1; ^#^*p* < 0.05 vs. R5 + morphine (M); ^¶^*p* < 0.05 vs. R5 + maraviroc (MVC); ^§^*p* < 0.05 vs. R5 + M + MVC; ^¥^*p* < 0.05 vs. R5 + M + MVC + naltrexone). (**a–b**) Modified and reprinted with permission from Arnatt et al. (2016). (**c–e**) Modified and reprinted with permission from El-Hage et al. (2013)

The cell type specific interactions between CCR5 and MOR were noted when using a bivalent ligand derivative of maraviroc linked to an opioid antagonist, naltrexone, with HIV-1 entry being significantly blocked in astrocytes but not microglia (El-Hage et al. 2013) (Fig. 5). Interestingly, maraviroc’s antiviral effects are completely negated in both astrocytes and microglia when morphine is present suggesting that maraviroc therapy may not be effective with opiate co-exposure. Importantly, unlike maraviroc, the bivalent compound blocked HIV entry in astrocytes irrespective of morphine treatment, while exacerbating HIV infectivity in morphine co-exposed microglia and revealing fundamental differences in the regulation of MOR and CCR5 expression in these glial types. Whereas, MOR and CCR5 expression appear to be similarly regulated in astrocytes, their expression patterns in microglia appear to be inversely correlated upon HIV and/or morphine exposure, with CCR5 being expressed at much higher levels than MOR (see El-Hage et al. 2013). The differential effects of the bivalent ligand in astrocytes compared to microglia might be due to the fact that the expression levels of MOR and CCR5 are differentially regulated by HIV in each of the cell types (El-Hage et al. 2013; Yuan et al. 2013; Arnatt et al. 2016).

The importance of CCR5 activation in glia, but not neurons, in mediating the neurotoxic effects of morphine-dependent MOR activation is further supported in a recent study demonstrating that the loss of CCR5 from glia (but not neurons) eliminated neurotoxicity due to Tat and morphine interactions (Kim et al. 2018). Similarly, short-duration (5 d) maraviroc pre-treatment also eliminated neurotoxicity and attenuated neuronal increases in [Ca^2+^]_i_ caused by Tat ± morphine (Kim et al. 2018). Selectively deleting either CCR5 (Kim et al. 2018) or MOR (Zou et al. 2011) from glia completely protects MSNs from morphine’s ability to exacerbate Tat neurotoxicity. However, deleting CCR5 from glia only revealed a paradoxical neuroprotective effect of morphine on Tat toxicity that is mediated by opioid receptors and appears to involve alterations in BDNF processing and signaling (Kim et al. 2018).

#### Enhanced Mature BDNF (mBDNF) Produced by CCR5 Deficient Glia is Neuroprotective

**Fig. 6:**
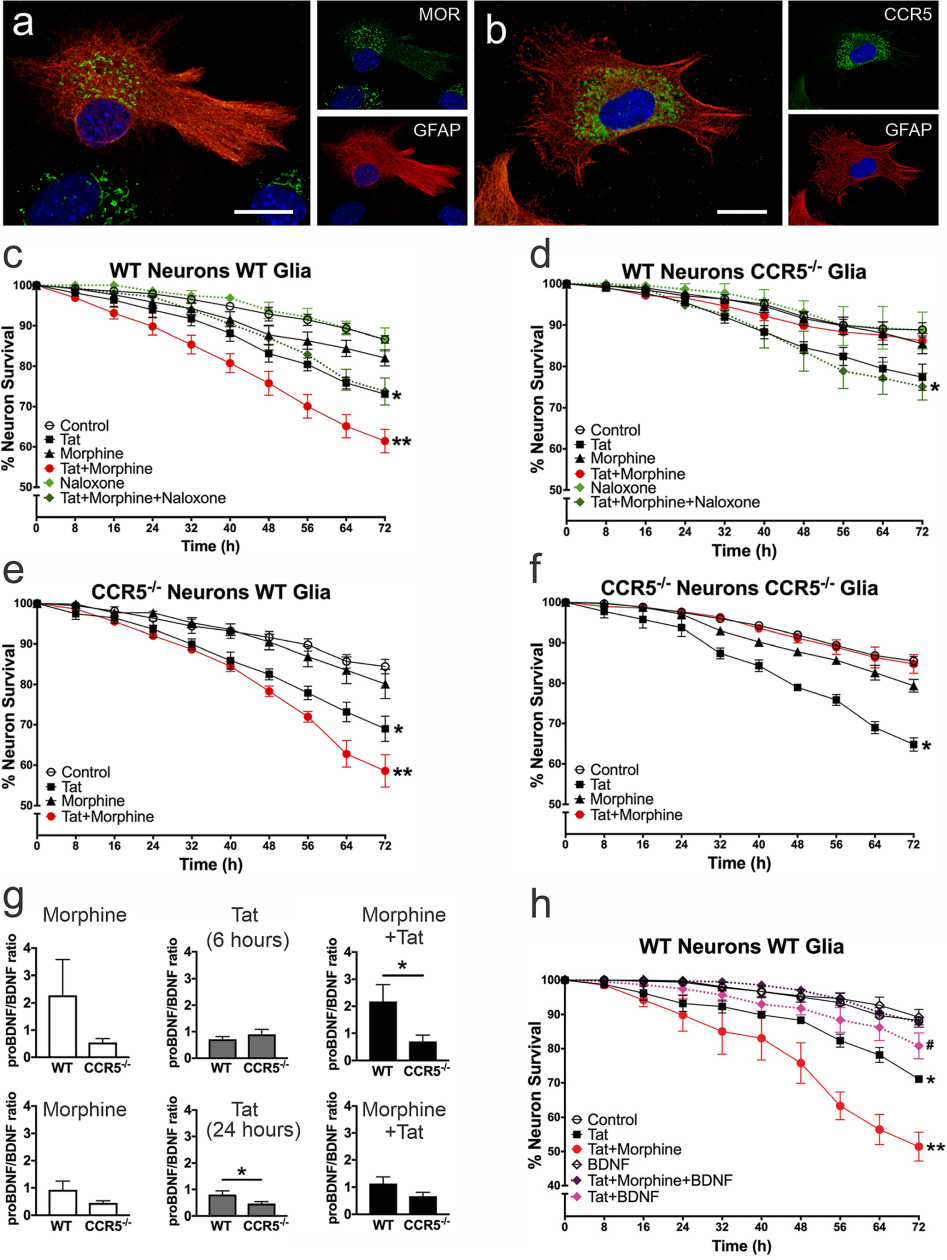
Role of CCR5 and BDNF in mediating HIV-1 Tat and morphine-induced interactive cytotoxicity in striatal medium spiny neurons (MSNs). A proportion of glial fibrillary acidic protein (GFAP)-immunolabeled striatal astrocytes display punctate patterns of μ-opioid receptor (MOR) (**a**) and CCR5 (**b**) (43.8 ± 2.4%) immunofluorescence—with some faint immunoreactivity extending into the cell processes; scale bars = 10 μm (**a-b**). HIV-1 Tat and morphine are no longer toxic to MSNs when CCR5 is deleted from glia (**c-f**). In C57BL/J wild-type mixed glia-MSN co-cultures, Tat is neurotoxic (*p = 0.001 vs. controls), and co-exposure to morphine enhanced Tat-induced toxicity over a 72-h period (**p < 0.001 vs. controls, *p* < 0.05 vs. Tat) and antagonized by naloxone (**c**). Naloxone or morphine by themselves had no effect on neuronal survival (**c**). In co-cultures with CCR5-deficit glia and wild-type neurons, exposure to Tat by itself is significantly toxic (*p < 0.001 vs. controls); however, the enhanced toxicity seen with combined morphine exposure was eliminated (**d**). Unexpectedly, morphine co-treatment entirely abolished the toxic effects of Tat, restoring MSN survival to control levels. Pre-treatment with naloxone re-established Tat toxicity, suggesting that the paradoxical protective effects of morphine are mediated by MOR (or perhaps another opioid receptor type) (**d**) (see Kim et al. 2018). The neurotoxic patterns seen in CCR5-deficient MSNs and wild-type glial co-cultures are similar to wild-type co-cultures (**e**). Co-cultures in which MSNs and glia are both deficient in CCR5 are similar to those in which CCR5 is only deficient in glia (**f**). CCR5 deletion alters the expression and processing of BDNF precursor (pro-BDNF) to mature (mBDNF) by mixed-glial cultures (**g**). BDNF is expressed by both astroglia and microglia; mBDNF is neuroprotective, while pro-BDNF can promote programmed cell death. mBDNF and pro-BDNF levels were analyzed in conditioned media from wild-type or CCR5-deficient mixed glia treated with Tat ± morphine after 6 h or 24 h to assess pro-BDNF and mBDNF levels. The proportion of pro-BDNF/mBDNF levels was significantly higher in wild-type compared to CCR5-null glia at 24 h (*lower row;* **g**), suggesting reduced neuronal support. Although morphine significantly decreased pro-BDNF in CCR5-deficient glia at both 6 h and 24 h compared to control levels (*not shown*), the pro-BDNF/mBDNF ratios were unaltered (*upper and lower rows;* **g**). By contrast, combined Tat and morphine significantly decreased the pro-BDNF/mBDNF ratio at 6 h, suggesting transient protection with CCR5 deficiency that was not fully sustained at 24 h (*p* = 0.17) (*p < 0.05, wild-type vs. CCR5-null) (**g**). Exogenous mBDNF is neuroprotective against combined Tat and morphine treatment (**h**). Wild-type, mixed glial-MSN co-cultures were treated with mBDNF and Tat, or combined Tat and morphine (represented by dotted survival curves). Tat alone was neurotoxic (*p < 0.05), and Tat was significantly worsened by co-exposing MSNs to morphine (**p < 0.0001). The addition of mBDNF (50 ng/ml; 72 h) fully protected MSNs against combined Tat and morphine toxicity, but only tended to protect (albeit not significantly) MSNs treated with Tat alone (^#^)(**h**). Overall, the results in **c-h** suggest (1) an important role for glial CCR5 in mediating HIV-1 and opiate neurotoxic interactions, (2) that CCR5 deficiency influences signaling through MOR, and (3) that CCR5 (and perhaps MOR) act via a BDNF intermediary to promote or obstruct neuronal survival (Kim et al. 2018). (**a-b**) Modified and reprinted with permission from Podhaizer et al. (2012). (**c-h**) Modified and reprinted from Kim et al. (2018), which is an open access article distributed under the terms of the *Creative Commons CC BY license*

Mature BDNF (mBDNF) activates tyrosine receptor kinase B (TrkB) and is neuroprotective, while the precursor to BDNF, pro-BDNF, binds p75^NTR^ and can activate cell death pathways. Based on findings of significant, reversible reductions in glially produced BDNF after exposure to HIV-infected cell supernatant ± morphine (Masvekar et al. 2014), altered BDNF processing in lymphocytes from PWH (Avdoshina et al. 2011), and following exposure to HIV-1 gp120 (Bachis et al. 2012), the pro-BDNF:mBDNF ratio was analyzed in supernatants from wild-type vs. CCR5-null striatal glial cultures exposed to Tat ± morphine for 6 or 24 h (Kim et al. 2018) **(**Fig. 6**)**. CCR5-deficiency reduced this ratio by over 2-fold in cells treated with Tat and morphine after 6 h **(**Fig. 6**)**, indicating a relative increase in mBDNF that may partially protect neurons in the CCR5-deficient glial environment.

Exogenous mBDNF treatment has been found to mimic the pro-survival effect of glial CCR5 deficiency against Tat ± morphine, and its neuroprotective effects have been supported in other neurodegenerative disease models (Cai et al. 2014; Xu et al. 2018). Collectively, the findings suggest that the loss of CCR5 may fundamentally change MOR signaling in HIV-exposed glia in a BDNF-dependent manner. Thus, overall the interaction of opioid and chemokine receptors, specifically MOR and CCR5, may alter the neuropathogenesis of HIV in a qualitatively unique manner not seen with either disorder alone.

### Does HIV Alter the Endogenous Opioid System?

Little is known about the effects of HIV on the endogenous opioid system and the extent to which HIV might disrupt the expression and function of opioid peptides and receptors, and vice versa. Because opiate drugs act exclusively by mimicking endogenous peptides and engaging opioid receptors, it is likely that endogenous opioids also interact with HIV to some extent to affect the pathogenesis of neuroHIV.

The endogenous opioid system comprises three originally described opioid receptors, MOR, KOR, and DOR and endogenous opioid peptide-expressing genes proopiomelanocortin (*POMC*), prodynorphin (*PDYN*), and proenkephalin (*PENK*) (Brownstein 1993; Trescot et al. 2008; Bodnar 2010; Pasternak and Pan 2013), as well as a fourth receptor (*OPRN1*) and peptide (nociceptin/orphanin FQ) family member. The endogenous opioid system has a fundamental role in pain regulation and has been implicated in the pathophysiology of various neurologic diseases (Nandhu et al. 2010; Sauriyal et al. 2011; Benarroch 2012) and in pain management (Bruehl et al. 2013).

Postmortem clinical studies indicate the endogenous opioid system is disrupted in neuroHIV (Gelman et al. 2012; Yuferov et al. 2014). Specifically, *OPRK1* mRNA is significantly upregulated in PWH (Yuferov et al. 2014) and in transgenic neuroHIV rodent models (Chang et al. 2007; Fitting et al. 2010b) potentially as a compensatory neuroprotective function in response to inflammatory processes in the presence of HIV infection (Yuferov et al. 2014). The upregulation of mRNA coding *OPRK1* is triggered by factors released by activated macrophages and glia and is supported by mechanistic studies in dorsal root ganglia (Puehler et al. 2006; Gabrilovac et al. 2012). Since leukocytes, including macrophages, can express β-endorphin and enkephalins, it is important to consider the potential influence of leukocyte-derived endogenous opioid peptides in neuroinflammation (Rittner et al. 2001). Granulocytes express about 10-fold higher levels of β-endorphin, a preferential MOR and lower affinity KOR endogenous ligand, than lymphocytes (Pallinger and Csaba 2008). Increases in β-endorphin expression by peripheral blood mononuclear cells (PBMCs) (Gironi et al. 2000; Gironi et al. 2003), coincide with inflammation and relapse in multiple sclerosis. Moreover, increases in inflammatory cytokines, such as interleukin-1β (IL-1β), have been demonstrated to differentially increase the expression of proenkephalin transcripts in primary astrocytes cultured from different brain regions (Ruzicka and Akil 1997) and increase IL-10-stimulated β-endorphin expression in cultured primary microglia (Wu et al. 2017). Interestingly, *OPRM1* mRNA levels do not differ between HIV+ and HIV− subjects (Yuferov et al. 2014).

*PENK* was downregulated in brain samples from 446 PWH compared to 67 HIV negative patients (Gelman et al. 2012). The subjects with HIV also expressed higher levels of interferon regulatory factor 1 (*IRF1*) transcripts. The idea that higher opioid peptide expression levels are neuroprotective has been supported in human studies and experimental animal models (Solbrig and Koob 2004; Sarkisyan et al. 2015; Nam et al. 2019) suggesting the reductions in *PENK* expression are deleterious.

The effects of HIV Tat on expression levels of opioid peptide and receptor levels depend on the individual CNS region involved as well as levels of *tat* transgene expression (Fitting et al. 2010b). For example, while *PDYN* mRNA levels were significantly reduced in the hippocampus and striatum of Tat-expressing mice, *POMC* was only significantly reduced by Tat induction in the striatum and *PENK* mRNA levels in the hippocampus were affected by chronic (but not acute) Tat exposure (Fitting et al. 2010b). Thus, HIV may alter the endogenous opioid system by modifying the expression of opioid peptides and their receptors in a brain- and cell-type specific manner. The consequences of HIV-1-dependent alterations in the endogenous opioid system to HAND are uncertain.

## Questions Remaining – Future Directions

### Modeling the Pharmacology of Opioid Self-Administration

Opiate self-administration as seen with addiction can have different CNS consequences than “steady-state” (e.g., continuous via a pump or time-release drug implant) exposure to the same drug (Kreek 1987, 2001; Kreek et al. 2002), and we predict the pharmacokinetic differences in opiate exposure will markedly impact neuroHIV progression. Differential effects based on “on-off” and “steady-state” drug administration schedules have been reported for the stress-responsive hypothalamic-pituitary-adrenal (HPA) axis, the endogenous opioid system, and the dopamine system (Kreek 1973; Kreek et al. 2002; George et al. 2012). Acute opiate exposure typically activates the HPA axis, corticotropin releasing factor, and peripheral steroidogenesis in a species-dependent manner (Koob and Kreek 2007; Cleck and Blendy 2008). Alternatively, chronic self-administration of short-acting opiates suppresses diurnal cortisol rhythmicity (Facchinetti et al. 1984; Vuong et al. 2010), while opiate withdrawal typically evokes HPA activation (Culpepper-Morgan and Kreek 1997; Kreek 2007; Paris et al. 2020). The daily, repeated bouts of relative withdrawal seen with opiate addiction cause sustained HPA activation, stress (Koob and Kreek 2007; Koob 2020), and immune suppression (Eisenstein 2019). Importantly, maintenance therapy with the long-acting drug methadone achieves steady-dose opiate levels and normalization of the HPA axis (Kreek 1973). Further, it is known that HIV infection significantly alters the HPA axis, due to CNS toxicity and cytokine production (Costa et al. 2000; George and Bhangoo 2013; Chrousos and Zapanti 2014).

Additionally, the nature of opiate exposure in the context of neuroHIV needs to be considered as it may induce different outcomes on neurotransmitter metabolism and gene expression. Specifically, the NAc shell demonstrates molecular and structural changes associated with intravenous heroin self-administration (Jacobs et al. 2005). Moreover, earlier studies have reported differential alterations in the turnover rates of various neurotransmitters for active versus passive morphine administration, including dopamine, serotonin, γ-aminobutyric acid (GABA), acetylcholine, aspartate, and glutamate during exposure to morphine (Smith et al. 1982, 1984). The disruptions were noticed specifically in brain regions involved in reinforcement processes, including the NAc, frontal cortex, and striatum, and encompassed increased dopamine and norepinephrine levels and turnover, which are central in opiate reward processes (Smith et al. 1982). Heroin abuse is known to downregulate dopaminergic activity in the NAc and may reflect a compensatory reduction in of dopamine biosynthesis in response to excessive dopaminergic stimulation resulting from chronic opiate exposure (Kish et al. 2001). Additionally, HIV is known to interfere with dopamine neurotransmission (Nath et al. 2000b; Gaskill et al. 2017) causing reductions in presynaptic dopamine terminals and dopamine transport in the striatum (Wang et al. 2004; Chang et al. 2008; Midde et al. 2012, 2015). The decline in dopamine function may exacerbate opioid abuse tendencies and drug-seeking behaviors as the rewarding effects of opioids are discounted by neuroHIV.

### Opioid Substitution Therapies and the Role of Selective/Biased Agonism in neuroHIV Pathogenesis

Although morphine, methadone, and buprenorphine all activate MOR, each can impart different signals through MOR, related to the nature and timing of their coupling to Gα, Gβγ, β-arrestin and/or regulators of G protein signaling (RGS), since each downstream effector couples into unique cell functions. Functional selectivity occurs at each opioid receptor type, and for most endogenous opioid peptides at all three receptor types (Gomes et al. 2020). Moreover, opioid receptors can be expressed on a subset of virtually every cell type in the CNS—with second messenger coupling to each opioid receptor type potentially being unique, cell-type specific, and context dependent. Thus, the “pluridimensional” (Galandrin and Bouvier 2006; Kenakin 2011; Costa-Neto et al. 2016) actions of any opiate at MOR are sufficiently complicated that it is not possible to predict whether, e.g., morphine, methadone or buprenorphine, would similarly effect any aspect of neuroHIV pathology without empirical testing. Despite their significant use as medication-assisted therapies for treating opioid addiction, few studies have directly compared commonly used opiate substitution therapies (Bell and Strang 2020), especially in relation to HIV (Khalsa et al. 2006; Choi et al. 2020).

Opioid substitution therapies significantly reduce the frequency of injection drug use (Kwiatkowski and Booth 2001; Pettes et al. 2010), decrease HIV transmission risk (MacArthur et al. 2012; Platt et al. 2016), and reduce drug-related mortality (Mathers et al. 2013) and the risk of opioid overdose (Volkow et al. 2014). Further, improved ARV outcomes among PWH have been reported with opioid substitution therapies, including the uptake and retention on ARV, medication adherence rates, and viral suppression (Low et al. 2016; Mukandavire et al. 2017). The two main medications used for opioid substitution therapy include methadone, a MOR full agonist, and buprenorphine, a MOR partial agonist and partial antagonist of KOR (Noble and Marie 2018). In comparison to methadone, buprenorphine has been shown to have fewer pharmacodynamic interactions with ARVs and causes less opioid withdrawal symptoms potentially due to its partial agonism on MOR, but also due to its high affinity and long duration of MOR binding (Walsh et al. 1994; McCance-Katz 2005; Whelan and Remski 2012). Further, differential proinflammatory and neurotoxic effects have been noted for various opioid treatments (Boland et al. 2014; Fitting et al. 2014b; Carvallo et al. 2015; Dutta and Roy 2015). In primary astrocytes, agonist-selective actions at MOR and KOR can be clearly demonstrated (Bohn et al. 2000; Belcheva et al. 2003; McLennan et al. 2008; Hahn et al. 2010), and we found that morphine, methadone, and buprenorphine differentially increase ROS and [Ca^2+^]_i_ alone or following Tat co-exposure (Fitting et al. 2014b). Morphine can enhance HIV-1-induced production of cytokines and specifically chemokines (El-Hage et al. 2008a; Dave 2012; El-Hage et al. 2014), while other opioids including methadone, oxycodone, buprenorphine, and DAMGO can decrease inflammatory function and decrease monocyte migration (Boland et al. 2014; Carvallo et al. 2015; Jaureguiberry-Bravo et al. 2016; Chilunda et al. 2019).

As most opiate drugs preferentially act via MOR, a potential explanation for differential interactive effects of opioids in the context of neuroHIV is the phenomenon of selective or “biased agonism”, such that different agonists can trigger distinct signaling events at the same receptor (Hauser et al. 2012). For example, coupling of MOR to Gα, Gβγ, and/or β-arrestin have been noted to differ depending on the MOR agonists involved (McPherson et al. 2010; Thompson et al. 2015; Burgueno et al. 2017). Physiologic outcomes of MOR activation in any cell type are determined by a bias for specific signaling pathways, the initial step of which is activation of G proteins and/or β-arrestin (Williams et al. 2013b; Violin et al. 2014; Suomivuori et al. 2020). The subcellular organization of GPCR signaling transduced by heterotrimeric G proteins and β-arrestin has been recently reviewed in detail (Eichel and von Zastrow 2018).

In the context of HIV, it has been shown that selective MOR agonists such as endomorphin-1, but not DAMGO or morphine, significantly increase HIV-1 replication in infected microglia (Peterson et al. 1999). This effect might be due to an apparent bias of endomorphin-1 towards arrestin recruitment and receptor phosphorylation, which was significantly correlated with agonist-induced internalization of MOR (McPherson et al. 2010). It is suggested that ligands that display bias towards G protein-mediated pathways and away from β-arrestin 2 recruitment may have improved therapeutic profiles against the development of tolerance and dependence/addiction (McPherson et al. 2010).

### Opioid Effects on Antiretroviral Efficacy within the CNS and Vice Versa

Opioid misuse has been linked to poor adherence to cART (Jeevanjee et al. 2014). However, adherence to ARV therapy improves after initiation of opioid substitution therapy (Nosyk et al. 2015; Low et al. 2016; Adams et al. 2020). Although better adherence can improve therapeutic outcomes in PWH, little information is currently available on the interaction between opioids or opioid substitution therapies and cART specifically within the CNS.

There are several known drug-drug interactions between opioids and ARVs that affect systemic concentrations. The partial opioid agonist, buprenorphine, is metabolized primarily by cytochrome P450 (CYP) 3A4 and 2C8. Both buprenorphine and its active metabolite, norbuprenorphine, are glucuronidated by UDP-glucuronosyltransferase (UGT) 1A1 and then excreted in bile. Several ARVs inhibit or induce these metabolic pathways. However, not all interactions are clinically relevant. The boosted protease inhibitor combination, atazanavir/ritonavir, inhibits CYP 3A4 and UGT 1A1, leading to increases in overall systemic exposure of buprenorphine and norbuprenorphine and also results in symptoms of opioid excess, such as increased sedation and impaired cognition (McCance-Katz et al. 2007). Dose adjustments of buprenorphine are recommended when initiating therapy with atazanavir to avoid symptoms of opioid excess. Methadone is a full opioid substrate with multiple metabolic pathways, including CYP 3A4, 2B6, 2C19, 2C9, and 2D6. Several pharmacokinetic interactions are reported between methadone and protease inhibitors. However, withdrawal symptoms are rare, and therefore, dose adjustments are not recommended (Bruce et al. 2006; Meemken et al. 2015). In contrast, efavirenz and nevirapine induce CYP 3A4, resulting in decreased systemic concentrations of methadone and the development of opioid withdrawal symptoms. To avoid opioid withdrawal, increased methadone dosing is recommended when either efavirenz or nevirapine therapy is initiated (Marzolini et al. 2000; Clarke et al. 2001; Meemken et al. 2015). Oxycodone metabolism is inhibited by lopinavir/ritonavir, increasing oxycodone concentrations as well as the self-reported drug effects (Nieminen et al. 2010; Feng et al. 2017).

The pharmacokinetic studies above focused on overall systemic exposure of drugs. Plasma concentrations, however, are not always accurate indicators of tissue exposure. Similarly, CNS drug exposure is often estimated based on drug concentrations within the CSF. However, CSF drug levels may not accurately predict brain concentrations. For many drugs with high efflux activities (e.g., substrates of P-gp), CSF tends to over-predict brain tissue concentrations (Liu et al. 2006; Friden et al. 2009; Kodaira et al. 2011, 2014). This could be due, in part, to differential expression of transporters at the blood-CSF barrier vs. BBB. In a study of the ARV drug amprenavir, concentrations of [^14^C]-amprenavir in CSF versus brain were 23.3 ± 11.2 and 3.33 ± 0.6 nCi/g, respectively, demonstrating overprediction of brain concentrations by CSF (Polli et al. 1999). These studies illustrate the high likelihood of misinterpreting drug brain penetration when using CSF as the surrogate marker. Therefore, direct measurement of brain tissue concentrations in animal models are likely to be more predictive of the interactive effects of ARVs and opioids on ARV and/or opioid brain exposure.

A few studies have investigated the impact of opioids and ARV administration on drug concentrations within the brain. One study investigated the impact of 5 d continuous exposure to morphine on ARV brain concentrations (dolutegravir, lamivudine and abacavir) and demonstrated that morphine exposure resulted in regionally specific decreases in the concentrations of select ARV drugs (Leibrand et al. 2019) and, furthermore, that the decreases in ARV concentrations (dolutegravir and abacavir) were likely due to increased efflux by the drug efflux transport protein, P-gp (Leibrand et al. 2019). Morphine alterations in P-gp within the brain could have wide reaching impact on other CNS active drugs.

HIV preferentially infects microglia and perivascular macrophages within the brain, although BMECs, astrocytes, and pericytes can also be infected (Kramer-Hammerle et al. 2005). Achieving optimal intracellular ARV concentrations are essential to suppress the infection. Few studies have examined whether ARV drugs differentially accumulate within different neural cell types and especially within cells of the neurovascular unit. Although nucleoside reverse transcriptase inhibitors (NRTIs) and non-nucleoside reverse transcriptase inhibitors (NNRTIs) are efficacious in inhibiting viral replication within monocyte-derived macrophages, only a few drugs within each ARV class can effectively inhibit viral replication within astrocytes (Gray et al. 2013), which could be a result of poor intracellular accumulation within astrocytes. In vitro studies have demonstrated darunavir and raltegravir intracellular concentrations to be approximately 100-fold lower (with higher EC_50_ values) in microglia than in PBMCs (Asahchop et al. 2017).

Another study measured intracellular concentrations of dolutegravir, tenofovir and emtricitabine in primary human astrocytes, microglia, pericytes and BMECs (Patel et al. 2019). Intracellular ARV concentrations were typically significantly higher in BMECs than in the other brain cell types. Dolutegravir achieved the highest relative concentrations within each cell type, whereas tenofovir accumulated the least (Patel et al. 2019). Furthermore, 24 h treatment with morphine significantly decreased intracellular accumulation of composite ARV concentrations, but only in astrocytes. In contrast, morphine exposure significantly increased the net accumulation of drugs within BMECs compared to controls. BMECs may sequester ARV drugs as a protective mechanism (Patel et al. 2019).

Using experimental data from SIV-infected, morphine-addicted macaques, mathematical modeling suggests that morphine exposure increases the proportion of cells with high susceptibility to SIV infection, at least in part, because of increased co-receptor expression (Vaidya et al. 2016). In addition to promoting a higher steady state viral loads and larger CD4 count declines, the model also predicts that morphine exposure results in the need for more efficacious ARV treatment than would be necessary for animals not exposed to morphine (Vaidya et al. 2016). Although the direct impact of morphine on ARV concentrations was not investigated, the study provides evidence supporting morphine’s negative impact on ARV efficacy.
